# Stimuli‐Directed Dynamic Reconfiguration in Self‐Organized Helical Superstructures Enabled by Chemical Kinetics of Chiral Molecular Motors

**DOI:** 10.1002/advs.201700613

**Published:** 2017-12-01

**Authors:** Jian Sun, Ruochen Lan, Yanzi Gao, Meng Wang, Wanshu Zhang, Ling Wang, Lanying Zhang, Zhou Yang, Huai Yang

**Affiliations:** ^1^ Department of Materials Science and Engineering University of Science and Technology Beijing Beijing 100083 P. R. China; ^2^ Department of Materials Science and Engineering College of Engineering Peking University Beijing 100871 P. R. China; ^3^ Artie McFerrin Department of Chemical Engineering Texas A&M University College Station TX 77843 USA; ^4^ Key Laboratory of Polymer Chemistry and Physics of Ministry of Education Peking University Beijing 100871 P. R. China

**Keywords:** adaptive materials, chemical kinetics, dynamic reconfiguration, helical superstructures, self organization

## Abstract

Dynamic controllability of self‐organized helical superstructures in spatial dimensions is a key step to promote bottom‐up artificial nanoarchitectures and functional devices for diverse applications in a variety of areas. Here, a light‐driven chiral overcrowded alkene molecular motor with rod‐like substituent is designed and synthesized, and its thermal isomerization reaction exhibits an increasing structural entropy effect on chemical kinetic analysis in anisotropic achiral liquid crystal host than that in isotropic organic liquid. Interestingly, the stimuli‐directed angular orientation motion of helical axes in the self‐organized helical superstructures doped with the chiral motors enables the dynamic reconfiguration between the planar (thermostationary) and focal conic (photostationary) states. The reversible micromorphology deformation processes are compatible with the free energy fluctuation of self‐organized helical superstructures and the chemical kinetics of chiral motors under different conditions. Furthermore, stimuli‐directed reversible nonmechanical beam steering is achieved in dynamic hidden periodic photopatterns with reconfigurable attributes prerecorded with a corresponding photomask and photoinduced polymerization.

## 1 Introduction

Development of stimuli‐responsive anisotropic architecture and nanostructures materials is a major driving force in fabricating reconfigurable and multifunctional devices for diverse applications.^1^, ^2^ As one of soft mesogenic architectures, liquid crystals (LCs) undoubtedly exhibit such fascinating tunable spatial anisotropy, resulting from the rearrangement of molecular alignment by external stimuli, such as chemical conversion, heat, pH, electric and magnetic field.^3^, ^4^, ^5^, ^6^, ^7^ Among numerous external stimuli, light is known to have superior advantages of remote, instant, and precise controllability in an uncontacted way. In this regard, many efforts have been devoted to advance photoresponsive molecular switches and motors in the realm of intelligent liquid‐crystalline materials.^8^, ^9^, ^10^, ^11^, ^12^, ^13^, ^14^ Light‐driven dynamic self‐organized helical superstructures, i.e., cholesteric liquid crystals (CLCs), could be obtained upon doping the chiral molecular motors into achiral LC hosts, where the orientation of helical axes is typically expressed as three configurations: (1) parallel to the substrate with a fingerprint texture (lying helixes) resulting from the periodical helical pitch (*p*) defined as the distance as the molecular rotation by 360° twist along the helical axis; (2) perpendicular to the substrate with a Grandjean texture (standing helixes) performing unique Bragg reflection of circularly polarized light at wavelength λ = *np*, where *n* is the average refractive index of LC; (3) spatial disorder alignment with a focal conic (FC) texture, which can strongly scatter incident light. Accordingly, light‐directed dynamic control over the helical axes as well as the helical pitch in one, two and three dimensions has been a burgeoning area for promising applications, including photodynamic photonic crystals, tunable mirrorless lasers, holographic microlenses, and light‐directed diffraction gratings.^15^, ^16^, ^17^, ^18^, ^19^, ^20^, ^21^

Photoinduced molecular isomerization in azobenzene derivatives has been extensively utilized to modulate the magnitude of order parameter in liquid‐crystalline system, resulting in the perturbation of molecular alignment and even phase transition, thus opening the door for optical‐pattern devices,^22^, ^23^, ^24^, ^25^, ^26^ microstructure photolithography,^27^, ^28^, ^29^ surface‐active topographies,^30^, ^31^, ^32^ and shape‐deformation actuators.^33^, ^34^, ^35^, ^36^, ^37^ Although the kinetic evolution of azo‐based LCs can be expedited by the anchoring memory effect in polymer‐stabilized mixture^38^, ^39^ or the push–pull effect in glassy elastomer,^37^, ^40^, ^41^ the inherently accelerative isomerization motion is still desirable, causing a variety of attractions to improve the essential action of isomerization compound with particular structures.^42^, ^43^, ^44^

**Figure 1:**
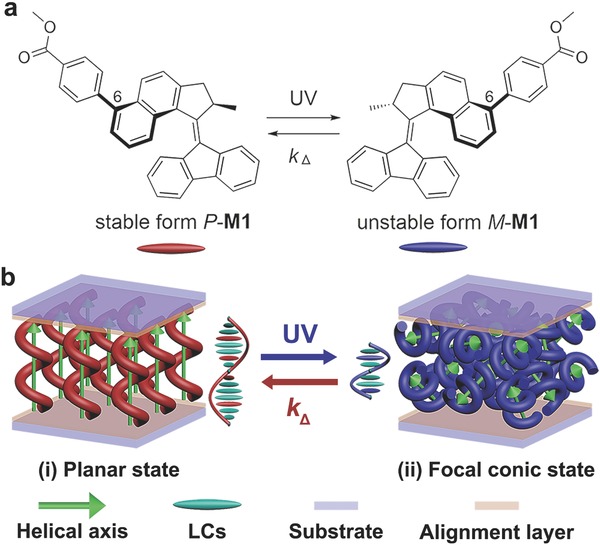
a) Photoisomerization and thermal inversion of chiral motors **M1** with methyl benzoate substituent at 6‐Position. b) Schematic mechanism of reversibly dynamic reconfiguration of motor‐doped self‐organized helical superstructures between (i) homogeneous helical axis at planar state with thermal relaxation and (ii) random helical axis at focal conic state upon UV irradiation.

Light‐driven molecular motors based on overcrowded alkenes pioneered by 2016 Nobel Prize Laureate B. L. Feringa have received vast attention in the last decade, where a repetitive rotation around the carbon–carbon olefinic bond as an axis was achieved by photocontrol of the dihedral angle between the “upper half” rotor and the “lower half” stator (**Figure** **1**a).^45^, ^46^ Due to the dynamic controllability of unidirectional rotation, the stereoselectively chiroptical inversion between the stable (*P*) form and the unstable (*M*) form is reversible with a variable half‐life by structural modification from 8 ns to 1.4 × 10^3^ years.^47^, ^48^, ^49^, ^50^ The resulting tunability of geometrical charity has been significantly investigated to create photoactiving chiral catalysts in solvents^51^, ^52^ and polar switchovers on solid surfaces.^53^, ^54^, ^55^ To further overcome the obstacle of Brownian motion, the guest‐host mesogenic hybrid systems make it possible to achieve long‐range ordered function in photoresponsive 1D photonic crystals with standing helixes^56^, ^57^, ^58^ and rotating microobjects on lying helixes.^59^, ^60^ Therefore, the kinetic analysis of chiral motor in liquid‐crystalline system is necessary to gain more bottom‐up information for the complex reconfiguration of artificial mesogenic construction, not only including the pitch variation of standing helixes and the in‐plane rotation of helical axes in lying helixes, but also taking account of the angular motion of helical orientation in spatial dimensions for emerging applications in areas of tunable photonics, intelligent energy‐saving, and controllable bioengineering.

Toward this end, the structural effect on the chemical kinetics of novel chiral overcrowded alkene in liquid‐crystalline media has been explored in this context. In contrast to the chiral overcrowded alkenes reported before,^48^, ^57^, ^61^ the new chiral motor **M1** in the present study was designed with structural modification on the rotor region (Figure 1a). As reported previously,^49^ the length of the rigid substituents at the 6‐position of naphthalene ring play an crucial role on the entropic barrier for rotation, but insignificantly affect the enthalpy of activation. Therefore, methyl benzoate was chosen as the substitution for the chiral motor **M1** because of its rigidity and resemblance to the rod‐like liquid crystal molecule that helps to improve the dopant‐host compatibility.^62^ To take advantage of the chemical kinetics of chiral motors, we report a facial control over the orientation of helical axes in self‐organized helical superstructures at the bistable local‐minimum free‐energy states, capable of light‐recorded spatial disorder (opaque state) in photochemical reaction and dark‐restored homogeneous alignment (transparent state) at thermally induced relaxation (Figure 1b). The reversible evolution of mesogenic reconfiguration was found to follow with the free energy fluctuation and the chemical kinetics of the doping motors in the photodynamic and thermodynamic processes. In this way, a periodic pattern can be encoded with alternately planar and FC configurations though a corresponding photomask, thereby enabling the dynamic controllability of optical diffraction device with an effectively prospective transmutation.

## 2 Results and Discussion

### 2.1 Chemical Kinetics of Structural Isomerization Reaction

**Figure 2:**
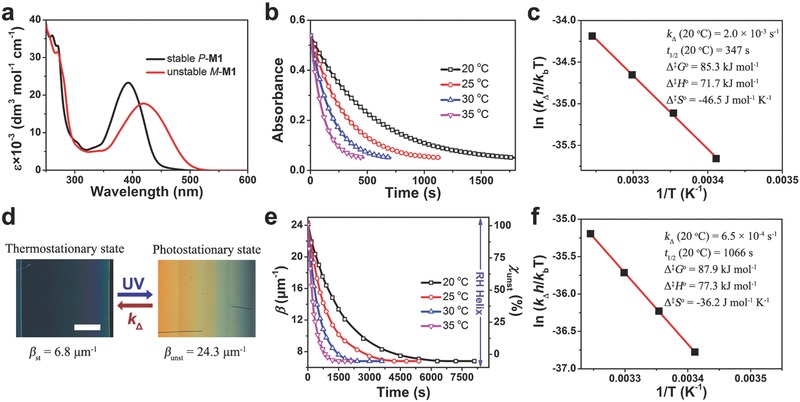
a) UV–vis absorption spectra of *P* **‐M1** and *M* **‐M1** in THF (40 µm) at room temperature. b) Unstable to stable absorption kinetics for **M1** at 450 nm at the specified temperatures. c) Erying plot for the thermodynamic inversion from unstable *M* **‐M1** to stable *P* **‐M1** isomer and the associated thermal kinetic parameters in THF. d) Crossed polarized optical texture of 1.0 wt% **M1** in SLC1717 in a wedge cell at the thermostationary state and the photostationary state. The scale bar represents 100 µm. e) HTP change of **M1** (1.0 wt% in SLC1717) and the corresponding molar fraction of *M* form during thermodynamic inversion at the specified temperatures. f) Erying plot for the thermodynamic inversion from unstable *M* **‐M1** to stable *P* **‐M1** isomer and the associated thermal kinetic parameters in LCs.

The light‐driven chiral molecular motor **M1** was prepared by a facile synthesis, and its chemical structure was characterized by ^1^H and ^13^C NMR spectroscopy, high‐resolution mass spectrometry, and elemental analysis (see Supporting Information). In general, the rotary motion of the motors was analyzed by UV–vis spectroscopy. Upon UV exposure (365 nm, 2.0 mW cm^−2^), the photoisomerization of chiral motor from the stable (*P*) form to the unstable (*M*) form occurred in tetrahydrofuran (THF), resulting in a bathochromic shift in the UV–vis absorption spectra from 392 to 419 nm (**Figure** **2**a). After cessation of UV light, the relaxation process from the photostationary state (PSS) to the thermostationary state (TSS) can be monitored by the time‐driven absorption spectra at 450 nm at several temperature (Figure 2b), fitting the first‐order chemical reaction model

(1) $$A(t)=A_{\infty }-\left(A_{\infty }-A_{0}\right)e^{-k_{\Delta }t}$$

where *A*_0_ is the photosaturated absorbance at the PSS, *A*_∞_ is the initial absorbance, and *k*_Δ_ is the thermal inversion rate. The half‐life (*t*_1/2_) and Gibbs free energy of activation (Δ^‡^ *G*°) at 20 °C can be obtained by means of the Eyring plot (Figure 2c), as well as the enthalpy of activation (Δ^‡^ *H*°) and entropy of activation (Δ^‡^ *S*°).

To investigate the chemical kinetics of chiral motor in mesophase, photoresponsive self‐organized helical superstructure by doping chiral motor **M1** (1.0 wt%, right‐handed) into commercially available achiral LC host SLC1717 (Slichem, *T*_N−I_ = 91.8 °C, *n*_e_ = 1.720, Δ*n* = 0.201) was examined by a transmission‐mode polarized optical microscopy (POM) with Grandjean–Cano wedge method.^63^ The helical twisting power (HTP, denoted as β) of chiral motor, where the positive and negative sign represent right‐handed and left‐handed helix, respectively, was monitored with respect to time at different temperature with equation

(2) $$\beta =\frac{1}{p\times c}$$

where *c* is the concentration of the chiral motor. As reported previously,^58^ the HTP is determined by the stereoselective photochemical equilibrium position of the chiral motor compounding with the *P* form and *M* form. Accordingly, the HTP value of chiral motor can be expressed by equation

(3) $$\beta =\beta_{\mathrm{unst}}\;\chi_{\mathrm{unst}}+\beta_{\mathrm{st}}\left(1-\chi_{\mathrm{unst}}\right)$$

where χ_unst_ is the molar fraction of *M* form, β_unst_ and β_st_ are the HTP values of *M* form and *P* form, representing at the PSS and the TSS at 20 °C, respectively (Figure 2d). Since the amount of the isomeric form of chiral motor in LCs is difficult to analyze by high‐performance liquid chromatography (HPLC) or NMR during the dynamic process, the thermal inversion step of the chiral motor can be followed by the χ_unst_ with respect to the percent change of HTP in time scale, fitting the first‐order model

(4) $$\chi_{\mathrm{unst}}(t)=\left|\frac{\beta (t)-\beta_{\mathrm{st}}}{\beta_{\mathrm{unst}}-\beta_{\mathrm{st}}}\right|=\chi_{\mathrm{unst},\mathrm{PSS}}\;e^{-k_{\Delta }t}$$

where χ_unst,PSS_ is the molar fraction of *M* form at the PSS, normalizing χ(0) = 1 and χ(∞) = 0 at 20 °C (Figure 2e). The thermodynamic relaxation for the χ_unst_ also conforms to the Eyring analysis within transition state theory at different temperature and the thermal kinetic parameters in LCs are given in Figure 2f.

To consider the major effect of methyl benzoate substituent in detail, the chemical kinetics of the overcrowded alkenes without the rod‐like substituent (**M2** and **M3**) was performed for the referenced analysis (Table S1, Supporting Information). It was found that motors **M1**, **M2,** and **M3** have the similar Δ^‡^ *H*° in the same media, whereas their Δ^‡^ *H*° in LC is increased compared to that in THF. It suggests that the Δ^‡^ *H*° of motor is mainly influenced by the frictional effect of viscous solvent in mesophase,^64^ rather than the steric hindrance in the fjord region or the electronic effect of polar group stemmed from the rod‐like substituent at the 6‐position.^49^, ^65^ On the other hand, the increasingly larger difference in Δ^‡^ *S*° between motor **M1** and the other overcrowded alkenes in LC, as compared to that in THF, is attributed to the pronounced intermolecular interaction between the rod‐substituent modified motor **M1** and the rod‐shape LC molecules in mesogenic system.^49^ As a consequence, the increasing Δ^‡^ *G*° of motor **M1** exhibited an obvious retardation of rotary motion in LC (*k*_Δ_ = 6.5 × 10^−4^ s^−1^), being three times as slow as that in THF (*k*_Δ_ = 2.0 × 10^−3^ s^−1^), while the other overcrowded alkenes remain the similar *k*_Δ_ in different media.^57^ These results indicate that the existence of the rod‐like substituent generates a noticeable enlargement of structural entropy effect in anisotropic LC host, distinguishing from the thermal relaxation process in isotropic organic solvent. It suggests that a relatively strong intermolecular interaction in the guest‐host system due to the resemblance of mesogenic groups. Additionally, the chemical dynamic isomerization motion of chiral molecular motor in self‐organized helical superstructures could be revealed by the helical pitch variation upon transition state theory with Eyring equation.

### 2.2 Dynamic Reconfiguration of Self‐Organized Helical Superstructures

**Figure 3:**
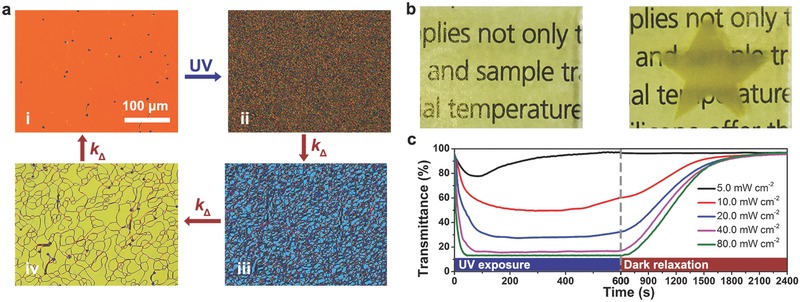
Stimuli‐induced configurational transition of **M1**‐doped CLC (8.5 wt% **M1** and 0.8 wt% S811 in SLC1717). a) POM images in reversible dynamic process: (i) planar reconfiguration, (ii) FC reconfiguration and (iii,iv) shrinking of the defect lines in dark relaxation. b) Photographs of the transparent state and the opaque state with a “star” pattern, respectively. c) Time‐resolved transmittance at 633 nm at different intensity of UV irradiation for 600 s and then dark relaxation at room temperature for 1800 s.

To further understand the influence of the chemical kinetics effect on the reconfiguration of self‐organized helical superstructures, photoresponsive CLC was prepared by doping 8.5 wt% chiral motor **M1** and 0.8 wt% S811 into SLC1717. The pitch variation from the initial state (*p*_0_ = 2.0 µm) to the PSS (*p*_PPS_ = 0.5 µm) was confirmed by Grandjean–Cano wedge method (Figure S5, Supporting Information). After being capillary‐filled into a 15 µm thickness cell coated with antiparallel alignment layer (polyvinyl acetate), the resulting CLC exhibited a Grandjean planar texture (**Figure** **3**a(i)). Upon UV exposure (80.0 mW cm^−2^), a FC configuration was developed (Figure 3a(ii)). A light‐scattering “star” pattern was obtained by photolithography with the corresponding photomask, and the unexposed area kept the light‐transparent state (Figure 3b). After removing UV source, the defect lines in FC domain gradually shrank with time and eventually disappeared, resulting in the reappearance of transparent state along the thermal inversion (Figure 3a(iii,iv)). The reversible dynamic switching of **M1**‐doped CLC was recorded by the time‐resolved transmittance spectroscopy at 633 nm under different UV intensity (Figure 3b), which can be explained by the theory of energy barrier in CLC.^66^ In terms of the director *n* of liquid crystal, the free energy density *F* is described by Frank–Oseen equation^67^, ^68^

(5) $$F=\frac{1}{2}K_{11}\;{\left(\nabla \cdot n\right)}^{2}+\frac{1}{2}K_{22}{\left[n\cdot \left(\nabla \times n\right)+q_{0}\right]}^{2}+\frac{1}{2}K_{33}{\left[n\times \left(\nabla \times n\right)\right]}^{2}$$

where the elastic constants *K*_ii_ and the deformation modes denote as *K*_11_ and ∇⋅n for splay, *K*_22_ and n⋅(∇×n) for twist, *K*_33_ and n×(∇×n) for bend, respectively, and wavevector *q*_0_ is twist deformation term only being available in cholesteric phase. Initially, the helical axes of the standing helixes homogeneously align perpendicular to the substrate under the boundary condition imposed by the alignment layer, displaying the transparent state at visible region (≈95% transmittance) (Figure 1b(i)). Hence, the free energy density is minimized in this equilibrium structure to only consider the twist term with *q*_0_ = 2π/*p*_0_. Under UV‐light‐induced isomerization of chiral molecular motor, a large number of defects and disclinations began to locally form in the fluctuant polydomain structures and the free energy density increased with the compression of helical pitch, which resulted in the metastable opaque phenomenon due to the mismatch of refractive indices among the disordered helical domains in 3D space (Figure 1b(ii)). As shown in Figure 3c, the photodynamic light‐scattering performance accelerated and strengthened along with increasing UV power because of the drop in helical pitch adapting to the excited photon‐density dependence on the photoisomerization ratio of chiral motor (Figure S11a, Supporting Information), as described in previous work.^58^ Importantly, since the elastic energy of defects, containing lines, loops, and knots, is positively correlated with the pitch length,^69^, ^70^, ^71^ an increasing energy barrier occurs between the free energy of the FC and planar reconfiguration in the photochemical reaction.^72^, ^73^ However, the light scattering performance spontaneously degenerated at the low excitation power density (<20.0 mW cm^−2^), as a result of the advantage of the surface anchoring energy of CLCs on the alignment layer over the low energy barrier with a large pitch length.^74^, ^75^ When the helical pitch was short enough at the high power density (>40.0 mW cm^−2^), the high energy barrier allowed the disordered helixes to be stabilized in FC domains at the PSS (Figure 1b(ii)). Upon ceasing UV irradiation followed by successive thermal inversion of chiral molecular motor, the height of the energy barrier falls down when the helical pitch is elongated. To minimize the free energy of the cholesteric system by penetrating the energy barrier of the large helical pitch, the helical axes rearrange to the uniform direction perpendicular to the substrate accompanying with the shrinking of the defect lines.^70^ The relaxing time required to reach the transparent state was almost constant at room temperature in the absence of UV exposure (Figure 3b). The thermal‐induced reconfiguration is mainly determined by the dark relaxation of helical pitch in the order of hundreds of seconds (Figure S11b, Supporting Information), beyond the characteristic reorganization time of LCs τ =*D* ^2^γ/*K*_22_ in the order of seconds, where *D* is the thickness of the cell and γ is the twist viscosity coefficient.^76^ These results demonstrate that the reversibly dynamic orientation of helical axes can be simply manipulated between homogeneousness and random in accordance with the minimum free energy of CLCs associated with the variation of helical pitch, which is triggered by the stable‐unstable isomerization of chiral motor to external stimuli for many cycles without obvious degradation (Figure S12, Supporting Information). Such kind of stimuli‐responsive film with the sensibility of UV intensity should find potential photonic applications, such as laser protection, self‐adaptive window, and biosensor.

**Figure 4:**
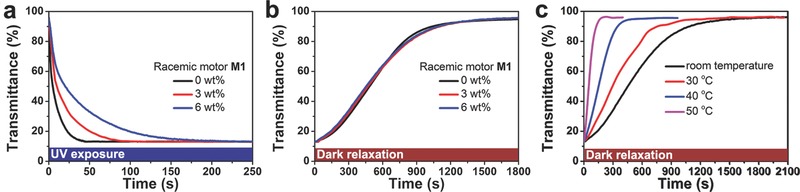
a) Photodynamic transmittance measurement (633 nm) of **M1**‐doped CLC with different concentration of racemic motor **M1** at 80.0 mW cm^−2^ and b) the corresponding thermodynamic process at room temperature. c) Thermodynamic transmittance measurement (633 nm) of **M1**‐doped CLC at different annealing temperature.

To verify the hypothetical mechanism, the kinetic evolution of the configurational transition for self‐organized helical superstructures was explored under different conditions. **Figure** **4**a shows the photodynamic processes of **M1**‐doped CLCs with different concentration of racemic motor **M1** for reconciling the free energy fluctuation with the pitch variation between *p*_0_ = 2.0 µm and *p*_PPS_ = 0.5 µm. The photoresponsive time required to reach the opaque state was found to become remarkably lengthened upon increasing the doping concentrations of racemic **M1**, where an increasing number of photoisomerization molecules needed to be excited and an damped intensity of UV light occurred due to the increasing absorptivity obeying the Beer–Lambert law.^77^ It indicates that the photodynamic reconfiguration of CLCs can be modulated by the photochemical isomerization–reabsorption process of chiral molecular motor. In addition, the dark relaxation achieves through the reversed helical pitch, which is determined by the first‐order thermal kinetics of motor but without correlation to the doping concentrations. In this manner, the thermodynamic processes of **M1**‐doped CLCs with different concentrations of racemic **M1** were invariant (Figure 4b), whereas the accelerative relaxation can be observed during rising temperature (Figure 4c), coinciding with the reverted rate of helical pitch under the control of *k*_Δ_ of motor **M1** in LC host (Figure 2e). Furthermore, the thermal relaxation can be readily tuned by the rotary motion of the chiral motor with different structural kinetics (Figure S13, Supporting Information). Thus, the micromorphology deformation of molecular alignment in self‐organized helical superstructure can be effectively dominated by the reversible stable–unstable isomerization of the chiral motor influenced by its chemical kinetics in the dynamic behavior.

### 2.3 Stimuli‐Directed Dynamic Diffraction Grating in Motor‐Doped CLCs

**Figure 5:**
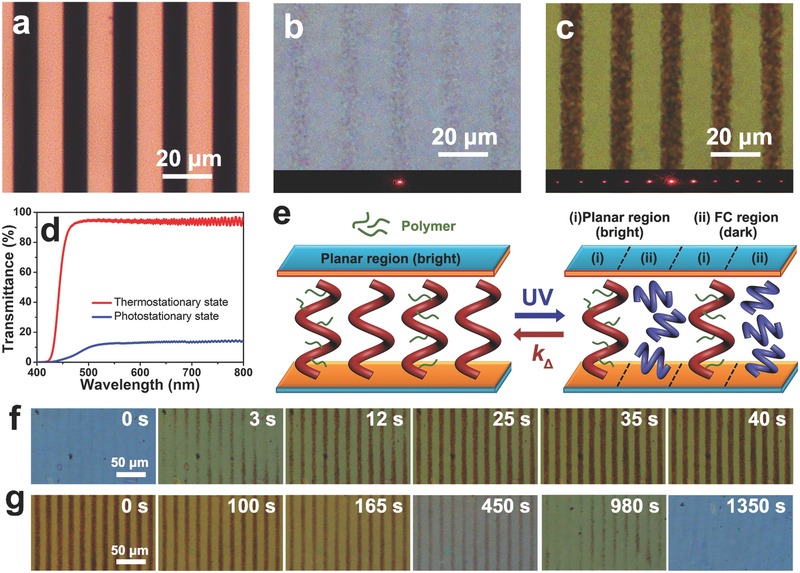
a) Optical microscopy image of the stripe photomask for fabricating the motor‐doped PSCLC grating. POM images of the **M1**‐doped PSCLC with b) homogeneous planar configuration at the TSS and c) binary pattern (alternate planar and FC configuration) at the PSS. Diffraction patterns of laser beam (λ = 633 nm) at the “off” and “on” states on underside. d) Transmittance spectra of the **M1**‐doped PSCLC at the TSS and the PSS, respectively. e) Schematic illustrations of reversibly dynamical PSCLC grating at the corresponding states. f) POM images of PSCLC grating during photodynamic evolution at 80.0 mW cm^−2^ and g) thermodynamic evolution at room temperature.

To delve potential applications of this stimuli‐driven reversible self‐organized helical superstructure, a diffraction grating of laser beam (λ = 633 nm) was investigated by the motor‐doped CLCs. In order to prevent the fluctuation of molecular diffusion on the topological structure in dynamic process, photopolymerisable liquid crystal monomer (20.0 wt%, C6M) and photoinitiator (1.0 wt%, Irgacure 784) were doped into **M1**‐doped CLC for fabricating the polymer‐stabilized cholesteric liquid crystals (PSCLCs) grating covered by a stripe photomask (**Figure** **5**a). The detailed photopolymerization process is given in Supporting Information (Figure S14, Supporting Information). After precrosslinking treatment upon 480 nm laser, the homogeneous planar configuration was observed in **M1**‐doped PSCLC and the probe beam could be detected at the diffraction “off” state (Figure 5b). Upon UV exposure (80.0 mW cm^−2^), a binary‐stripe pattern was developed within 50 s, consisting of alternate planar and FC configurations, to effectively turn on the diffraction pattern of the probe beam with a grating period of ≈20 µm (Figure 5c), and the optical transmittance of **M1**‐doped PSCLC sharply decreased from 95% to 12% at the PSS (Figure 5d). As schematically depicted in Figure 5e, the prepolymerization helical superstructures were always stabilized to be the standing helixes as the bright regions by the crosslinking‐aggregation polymer network, whereas the adjacent helical superstructures without the anchoring limitation of polymer network would be photodynamically switched to the disordered helixes as the black regions, and subsequently reverted to the bright state followed by the thermal inversion under the stimuli‐directed helical pitch. Promisingly, the optically controllable beam‐steering capability was achieved by the formation of light‐recorded periodic topography, resulting from the asymmetry of luminous flux between the bright and dark regions.

**Figure 6:**
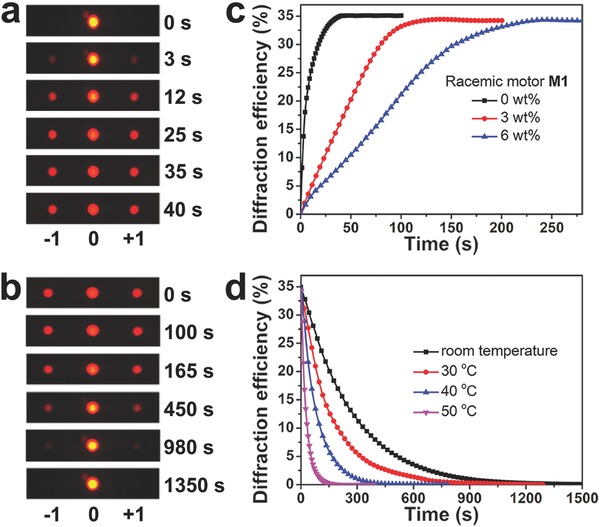
a) Temporal evolution of diffraction pattern for **M1**‐doped PSCLC grating in photodynamic process and b) thermodynamic process. c) The rise response time of first‐order diffraction efficiency for **M1**‐doped PSCLC with different concentration of racemic motor **M1** at 80.0 mW cm^−2^. d) The decay response time of **M1**‐doped PSCLC at different annealing temperature.

The chemical structure‐kinetics effect of chiral motor on the dynamical diffraction evolution of the motor‐doped PSCLC grating was further investigated. **Figure** **6**a,b show the time‐recorded diffraction patterns of the **M1**‐doped PSCLC grating upon UV exposure (80.0 mW cm^−2^) and thermal relaxation at room temperature, matching up to the temporal reconfiguration of **M1**‐doped PSCLC grating as shown in Figure 5f,g. The first‐order diffraction beam gradually appeared within 40 s and its diffraction efficiency increased to ≈35% being transferred from the energy of zeroth‐order diffraction, where the diffraction efficiency is defined as a ratio of the intensity of the order‐diffracted beam to the total intensity before UV irradiation. The reverted process was achieved within 1350 s. As excepted, the first‐order diffraction angle was constantly measured to be 1.81°, which is well‐fitting to the theoretical value sin θ_m_ = *mλ*/*Λ*, where θ_m_ is the diffraction angle, *m* is the diffraction order, λ is the laser wavelength, and *Λ* is the grating period.^78^ The rise response times of the first‐order diffraction for the **M1**‐doped PSCLC took a delay when increasing the concentration of racemic motor **M1** (Figure 6c). It is associated with the photodynamic transition of **M1**‐doped CLCs from the planar to the FC configuration as shown in Figure 4a, resulting from the photoisomerization–reabsorption interrelation of molecular motors only with regard to the doping concentration. Figure 6d shows that the decay response variation of the first‐order diffraction for **M1**‐doped PSCLC grating during annealing at different temperature, which is agreed with the thermal isomerization rate of chiral motor **M1** in liquid crystal host, as shown in Figure 4c. Besides, the modulated erasure of diffraction gratings for motor‐doped PSCLCs was demonstrated by the chemical kinetics of chiral motors **M1**–**M3** with the corresponding modified structure (Figure S15, Supporting Information).

## 3 Conclusions

In summary, a new light‐driven chiral overcrowded alkene rotor with structural modification have been judiciously designed and synthesized. Its chemical kinetic analysis shows that the higher entropic barrier for the rotary motion in anisotropic LC host can be attributed to the enhanced intermolecular interaction between the rod‐like substituent and structurally similar rod‐shape LC molecules, resulting in a different degree of chemical kinetics in comparison with the situation in isotropic organic solvent. Furthermore, stimuli‐induced dynamic self‐organized helical superstructures loaded with the chiral molecular motor was fabricated and their capability of angular motion of helical superstructures was demonstrated. The light‐pattern response is majorly determined by the concentration of chiral motor and the UV‐exposure intensity, and the thermal rearrangement of helical superstructures would confirm to the rotary motion of chiral motor with different chemical structure effect and annealing temperature. Such stimuli‐directed dynamic cholesteric superstructures were further used to enable reversible 1D beam steering switching between “on” and “off” states by the light‐recorded stripe lithography. The temporal evolution of the 1D diffraction patterns also fed back the dynamic reconfiguration of helical superstructures at an anticipative rate obeying the chemical kinetics of chiral motor. This research offers insight into the realization of complex micromorphology reconfiguration in stimuli‐induced anisotropic architectures, linking chemical kinetics of molecular isomerization and physical deformation of highly ordered nanostructure in dynamic behavior. It is possible to significantly advance new direction for further intelligent applications in the spatially and temporally high‐performance photonic action.

## 4 Experimental Section

Experimental details are described in the Supporting Information.

## Conflict of Interest

The authors declare no conflict of interest.

## Supporting information

Supplementary
