# Supplementary material for: Stimuli‐Directed Dynamic Reconfiguration in Self‐Organized Helical Superstructures Enabled by Chemical Kinetics of Chiral Molecular Motors
Source: Adv Sci (Weinh). 2017 Dec 1;5(2):1700613. doi: 10.1002/advs.201700613 (PMC5827101; doi:10.1002/advs.201700613)
Supplement: Supplementary file 1 — Supplementary [file ADVS-5-1700613-s001.pdf]

Copyright WILEY-VCH Verlag GmbH & Co. KGaA, 69469 Weinheim, Germany, 2017.

ADVANCED SCIENCE Open Access

Supporting Information

for *Adv. Sci.,* DOI: 10.1002/advs.201700613

Stimuli-Directed Dynamic Reconfiguration in Self-Organized Helical Superstructures Enabled by Chemical Kinetics of Chiral Molecular Motors

Jian Sun, Ruochen Lan, Yanzi Gao, Meng Wang, Wanshu Zhang, Ling Wang, Lanying Zhang, Zhou Yang, and Huai Yang**

Copyright WILEY-VCH Verlag GmbH & Co. KGaA, 69469 Weinheim, Germany, 2017.

Supporting Information

Stimuli-Directed Dynamic Reconfiguration in Self-Organized Helical Superstructures Enabled by Chemical Kinetics of Chiral Molecular Motors

Jian Sun, Ruochen Lan, Yanzi Gao, Meng Wang, Wanshu Zhang, Ling Wang, Lanying Zhang, Zhou Yang, Huai Yang**

Content

1. **Experimental details**
2. **Synthesis of overcrowded alkene M1-M3**
3. **Kinetic analysis of motors M1-M3 in THF**
4. **Photo-induced and thermo-induced chiroptical variation of chiral motors M1-M3 in cholesteric liquid crystals (CLCs)**
5. **Dynamic analysis of chiral motors M1-M3 in CLCs**
6. **Fabrication and characterizations of motor-doped CLCs and polymer-stabilized CLCs (PSCLCs)**
7. **^{1}H NMR and ^{13}C NMR spectra**
8. **References**

1. Experimental details

All starting chemicals, solvents and reagents were purchased from Sigma-Aldrich, TCI or J&K. Analytical thin-layer chromatography (TLC) was performed with aluminum sheets coated on Merck silica gel 60 F254. Column chromatography was carried out on silica gel (200-300 mesh). ^{1}H and ^{13}C spectra were recorded on a Bruker 400 MHz spectrometer. Chemical shifts (δ) are denoted in parts per million (ppm) relative to the residual solvent peak (TMS, ^{1}H δ = 0.00, ^{13}C δ = 0.00; $CDCl_3$, ^{1}H δ = 7.26, ^{13}C δ = 77.16). The splitting patterns of peaks are designated as follow: s (singlet), d (doublet), t (triplet), m (multiplet), br (broad), or dd (doublet of doublets). HRMS spectra were obtained with an ThermoFisher Q Exactive GC-MS/MS System. Elemental analysis was performed by Elementar Analysensysteme GmbH vario EL. UV/Vis measurements were taken by a Perkin Elmer Lambda 950 spectrophotometer. Textures and disclination line of cholesteric liquid crystals (CLCs) in Grandjean-Cano wedge cell (KCRK-07, tan θ = 0.0196, EHC) were observed by polarized optical microscopy (POM, Axio Scope A1 pol, Zeiss). The UV light (365 nm) irradiation was carried out with a LED lamp (FUV-6BK, Bangwo Elec. Technologies Co., Ltd.). The 480 nm and 633 nm laser beams with 8 ns pluse width and the 10 Hz repetition frequency, derived from a Nd: YAG second harmonic generation pulse laser (Spilight 1000 OPO Broadband) were used as the pump light. The intensity of the order-diffracted beam was monitored by a photodiode. A commercial nematic liquid-crystal host SLC1717 (Slichem, $T_{N\text{-}I}$ = 91.8 ^{o}C, n_e = 1.720, Δn = 0.201), chiral dopant S811 (Merck), photo-polymerisable liquid-crystalline monomer C6M (Lab Synthesized)[1] and visible-light sensitive photoinitiator Irgacure 784 (TCI Co., Ltd.) were used in the study (Figure S7).

2. Synthesis of overcrowded alkene M1-M3

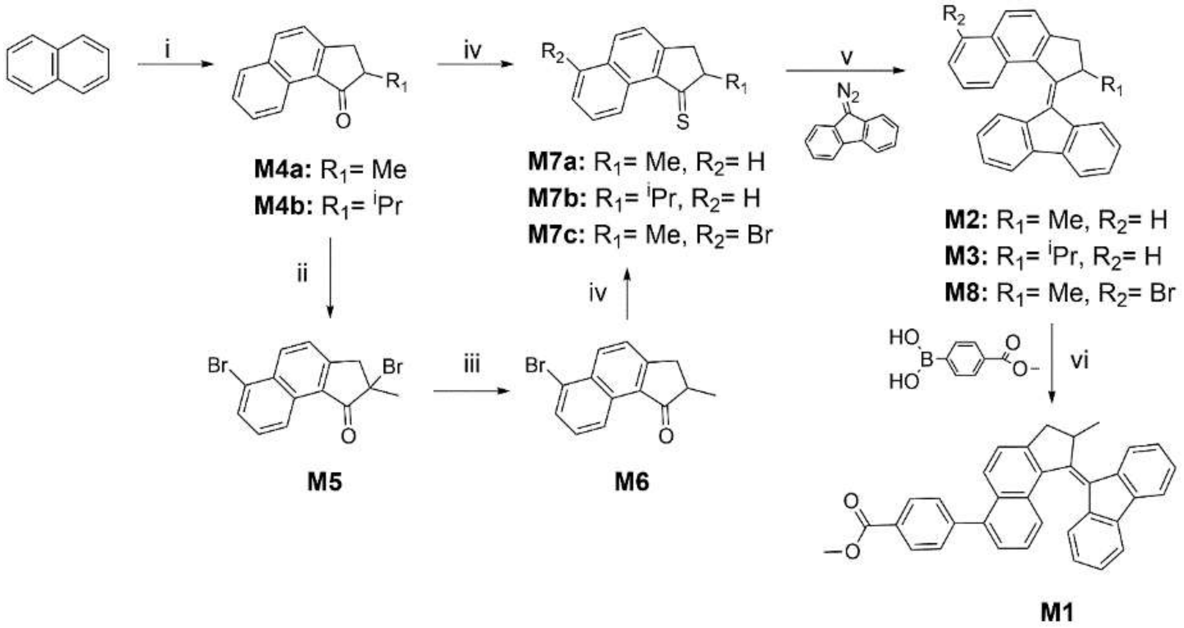

Scheme S1. Synthesis of motor **M1-M3**. Reagents and conditions: (i) methacrylic acid or 2-isopropylacrylic acid, PPA, 110 °C, 4h; (ii)$AlCl_3$, Br_2, AcOH, 40 °C, 2d; (iii) NaI, TMSCI, CH_3CN, 12h; (iv) Lawsson's reagent, toluene, 90 °C, reflux, 4h; (v) PPh_3, toluene, 90 °C, reflux, 12h; (vi) $Pd(PPh_3)_4$, Ag_2O, Cs_2CO_3, THF, reflux, 12h.

2-methyl-2,3-dihydro-1H-cyclopenta[a]naphthalen-1-one (M4a)

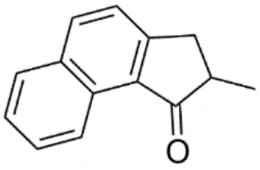

To polyphosphoric acids (250 ml) at 80 °C was added naphthalene (3.20 g, 25.0 mmol). The mixture was heated to 110 °C under mechanical stirrer, after which methacrylic acid (3.23 g, 37.5 mmol) was added and stirred for 4 h. After cool to 70 °C, the mixture was poured into ice water and stirred for an additional 3 h. The mixture was extracted with CH_2Cl_2 (3 x 50 mL). The organic extracts were washed with $NaHCO_3$ (saturated aqueous solution, 50mL), brine and water and dried on $MgSO_4$. The solvent was removed *in vacuo* and the crude residue was purified by column chromatography (SiO_2, pentane: CH_2Cl_2 = 3:1) to yield **M4a** as light yellow liquid (2.10 g, 41%) ^{1}HNMR (400 MHz, $CDCl_3$): δ = 9.15 (d, J = 10.4 Hz, 1H), 8.02

(d, J = 11.1 Hz, 1H), 7.87 (d, J = 10.8 Hz, 1H), 7.66 (t, J = 9.3 Hz, 1H), 7.54 (t, J = 10.1 Hz, 1H), 7.48 (d, 1H), 3.46 (dd, J = 24.2, 10.8 Hz, 1H), 2.90 – 2.60 (m, 2H), 1.37 (d, J = 9.7 Hz, 3H).

2-isopropyl-2,3-dihydro-1H-cyclopenta[a]naphthalen-1-one (M4b)

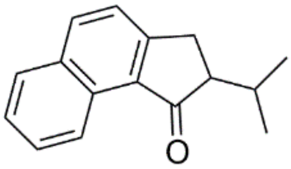

To polyphosphoric acids (250 ml) at 80 ^{o}C was added naphthalene (3.20 g, 25.0 mmol). The mixture was heated to 110 ^{o}C under mechanical stirrer, after which 2-isopropylacrylic acid (4.28 g, 37.5 mmol) was added and stirred for 4 h. After cool to 70 ^{o}C, the mixture was poured into ice water and stirred for an additional 3 h. The mixture was extracted with CH_2Cl_2 (3 x 50 mL). The organic extracts were washed with $NaHCO_3$ (saturated aqueous solution, 50mL), brine and water and dried on $MgSO_4$. The solvent was removed *in vacuo* and the crude residue was purified by column chromatography (SiO_2, pentane: CH_2Cl_2 = 5:1) to yield **M4b** as light yellow liquid (1.79 g, 32%) ^{1}HNMR (400 MHz, $CDCl_3$): δ = 9.18 (d, J = 11.1 Hz, 1H), 8.03 (d, J = 11.2 Hz, 1H), 7.89 (d, J = 10.1 Hz, 1H), 7.67 (t, J = 9.3 Hz, 1H), 7.54 (dd, J = 14.8, 11.0 Hz, 2H), 3.22 (dd, J = 23.7, 10.1 Hz, 1H), 3.01 (dd, J = 23.7, 4.6 Hz, 1H), 2.90 – 2.69 (m, 1H), 2.62 – 2.39 (m, 1H), 1.10 (d, J = 9.2 Hz, 3H), 0.81 (d, J = 9.1 Hz, 3H).

2,6-dibromo-2-methyl-2,3-dihydro-1H-cyclopenta[a]naphthalen-1-one (M5)

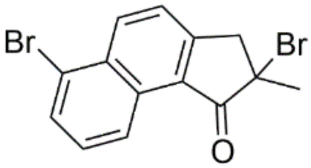

Compound **M5** was synthesized according to literature procedure.[2] **M5** was obtained as orange solid (900 mg, 50%). ^{1}HNMR (400 MHz, $CDCl_3$): δ = 9.14 (d, J = 8.4 Hz, 1H), 8.59

(d, *J* = 8.7 Hz, 1H), 7.89 (d, *J* = 7.5 Hz, 1H), 7.66 – 7.45 (m, 2H), 3.92 (d, *J* = 18.6 Hz, 1H), 3.62 (d, *J* = 18.6 Hz, 1H), 2.02 (s, 3H).

6-bromo-2-methyl-2,3-dihydro-1H-cyclopenta[a]naphthalen-1-one (M6)

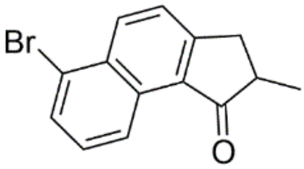

Compound **M6** was synthesized according to literature procedure.[2] **M6** was obtained as white solid (800 mg, 85%). ^{1}HNMR (400 MHz, $CDCl_3$): δ 9.17 (d, *J* = 8.4 Hz, 1H), 8.47 (d, *J* = 8.7 Hz, 1H), 7.83 (d, *J* = 7.5 Hz, 1H), 7.58 (d, *J* = 8.7 Hz, 1H), 7.54 – 7.43 (m, 1H), 3.48 (dd, *J* = 18.2, 8.1 Hz, 1H), 3.00 – 2.69 (m, 2H), 1.38 (d, *J* = 7.3 Hz, 3H).

2-methyl-2,3-dihydro-1H-cyclopenta[a]naphthalene-1-thione (M7a)

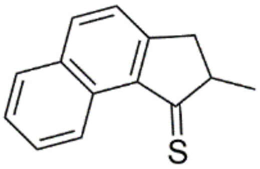

To a solution of **M4a** (0.98 g, 5.0 mmol) in toluene (100 mL) was added Lawsson's reagent (4.04 g, 10.0 mmol). The mixture was stirred for 4 h at 90 ^{o}C, after which it was allowed to cool down and filtered at . The solvent was removed *in vacuo* and the crude residue was purified by column chromatography (SiO_2, pentane: ethyl acetate = 10:1) to yield **M7a** as dark blue oil (637 mg, 60%). The obtained **M7a** was used to the next reaction immediately.

2-isopropyl-2,3-dihydro-1H-cyclopenta[a]naphthalene-1-thione (M7b)

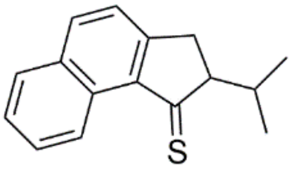

Compound **M7b** was synthesized according to the general procedure (iv). **M7b** was obtained as dark blue oil (841 mg, 70%). The obtained **M7b** was used to the next reaction immediately.

6-bromo-2-methyl-2,3-dihydro-1H-cyclopenta[a]naphthalene-1-thione (M7c)

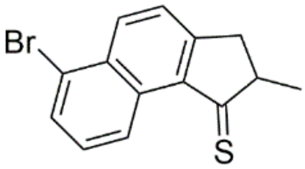

Compound **M7c** was synthesized according to the general procedure (iv). **M7c** was obtained as dark blue oil (2.0 g, 41%) The obtained **M7c** was used to the next reaction immediately.

9-(2-methyl-2,3-dihydro-1H-cyclopenta[a]naphthalen-1-ylidene)-9H-fluorene (M2)

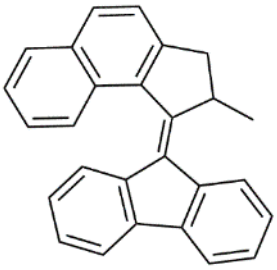

A solution of **M7a** (700 mg, 3.3 mmol) and 9-diazo-9H-fluorene (769 mg 4.0 mmol) in toluene (100 mL) was stirred for 1 h at 90 ^{o}C. PPh_3 (918 mg, 3.5 mmol) was added to the mixture and reaction continued for 4h. The stirred solution was allowed to cool down to room temperature and MeI (568 mg, 4.0 mmol) was added to remove the excess of PPh_3 for 6h. After filtering the deposit, the solvent was removed *in vacuo* and the crude residue was purified by column chromatography (SiO_2, pentane: CH_2Cl_2 = 10:1) to yield **M2** as yellow solid (625 mg, 55 %). ^{1}H NMR (400 MHz, $CDCl_3$): δ = 7.99 (dd, J = 5.6, 3.2 Hz, 1H), 7.92 (t, J = 8.9 Hz, 2H), 7.85 (dd, J = 5.6, 3.1 Hz, 1H), 7.76 (dd, J = 7.9, 4.7 Hz, 2H), 7.57 (d, J = 8.2 Hz, 1H), 7.46 (t, J = 7.4 Hz, 1H), 7.39 (dd, J = 5.7, 3.1 Hz, 2H), 7.31 (t, J = 7.6 Hz, 1H), 7.21 (t, J = 7.4 Hz, 1H), 6.80 (t, J = 7.2 Hz, 1H), 6.72 (d, J = 7.9 Hz, 1H), 4.47 – 4.24 (m, 1H), 3.57 (dd, J = 15.0, 5.6 Hz, 1H), 2.76 (d, J = 15.0 Hz, 1H), 1.38 (d, J = 6.7 Hz, 3H); ^{13}C NMR

(100 MHz, $CDCl_3$): δ = 151.19, 147.47, 140.15, 139.88, 139.61, 137.18, 136.42, 132.69, 130.92, 130.50, 129.89, 128.72, 127.57, 126.98, 126.94, 126.63, 125.94, 125.36, 124.10, 124.05, 119.73, 119.00, 45.57, 42.03, 19.36. HRMS (ESI) calcd for $C_{27}H_{20}Na^+$ 367.1463, found 367.1451. Anal. Calcd for $C_{27}H_{20}$: C 94.15, H 5.85; found: C 94.19, H 5.81. Chiral resolution of **M2** was achieved by preparative SFC, using a chiral ChiralCN OD column (Guangzhou Research & Creativity Biotechnology Co., Ltd.) in CO_2: EtOH = 4:1, the enantiomeric excess (*e.e.*) = 99.7 %.

9-(2-isopropyl-2,3-dihydro-1H-cyclopenta[a]naphthalen-1-ylidene)-9H-fluorene (M3)

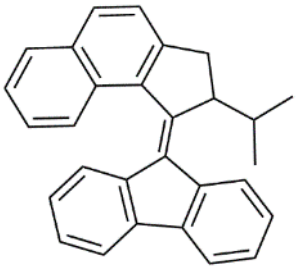

Compound **M3** was synthesized according to the general procedure (v). **M3** was obtained as yellow solid (599 mg, 52%). ^{1}H NMR (400 MHz, $CDCl_3$): δ = 8.04 – 7.96 (m, 1H), 7.91 (dd, *J* = 15.1, 8.2 Hz, 2H), 7.87 – 7.82 (m, 1H), 7.76 (dd, *J* = 7.6, 5.5 Hz, 2H), 7.54 (d, *J* = 8.2 Hz, 1H), 7.45 (t, *J* = 7.5 Hz, 1H), 7.39 (dd, *J* = 5.5, 3.2 Hz, 2H), 7.32 (t, *J* = 7.6 Hz, 1H), 7.20 (t, *J* = 7.3 Hz, 1H), 6.77 (t, *J* = 7.6 Hz, 1H), 6.70 (d, *J* = 7.9 Hz, 1H), 4.21 (t, *J* = 5.4 Hz, 1H), 3.41 (dd, *J* = 15.4, 5.8 Hz, 1H), 2.97 (d, *J* = 15.4 Hz, 1H), 2.26 (s, 1H), 1.15 (d, *J* = 7.0 Hz, 3H), 0.58 (d, *J* = 6.7 Hz, 3H); ^{13}C NMR (100 MHz, $CDCl_3$): δ = 150.49, 148.23, 140.28, 139.77, 139.35, 137.98, 137.11, 132.48, 130.99, 130.76, 129.11, 128.68, 127.29, 126.89, 126.84, 126.74, 126.63, 125.91, 125.82, 125.26, 124.26, 123.20, 119.65, 118.81, 56.33, 36.25, 31.16, 20.97, 18.58. HRMS (ESI) calcd for $C_{29}H_{24}Na^+$ 395.1775, found 395.1784. Anal Calcd for $C_{29}H_{24}$: C 93.51, H 6.49; found: C 93.54, H 6.46. Chiral resolution of **M3** was achieved by

preparative HPLC, using a chiral ChiralCN OD column (Guangzhou Research & Creativity Biotechnology Co., Ltd.) in toluene: EtOH = 1:1, the enantiomeric excess (*e.e.*) = 99.1 %.

9-(6-bromo-2-methyl-2,3-dihydro-1H-cyclopenta[a]naphthalen-1-ylidene)-9H-fluorene (M8)

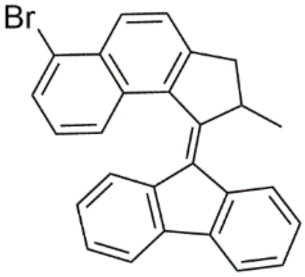

Compound **M8** was synthesized according to the general procedure (v). **M8** was obtained as orange solid (1.4 g, 48%). ^{1}H NMR (400 MHz, $CDCl_3$): δ = 8.35 (d, *J* = 8.5 Hz, 1H), 7.95 (dd, *J* = 5.6, 3.2 Hz, 1H), 7.81 (dd, *J* = 5.6, 3.2 Hz, 1H), 7.78 (d, *J* = 8.4 Hz, 1H), 7.72 (d, *J* = 7.4 Hz, 2H), 7.63 (d, *J* = 8.5 Hz, 1H), 7.37 (dd, *J* = 5.7, 3.1 Hz, 2H), 7.19 (t, *J* = 7.4 Hz, 1H), 7.14 – 7.06 (m, 1H), 6.79 (t, *J* = 7.4 Hz, 1H), 6.66 (d, *J* = 7.9 Hz, 1H), 4.43 – 4.23 (m, 1H), 3.55 (dd, *J* = 15.2, 5.6 Hz, 1H), 2.73 (d, *J* = 15.2 Hz, 1H), 1.35 (d, *J* = 6.7 Hz, 3H).

methyl 4-(1-(9H-fluoren-9-ylidene)-2-methyl-2,3-dihydro-1H-cyclopenta[a]naphthalen-6-yl)benzoate (M1)

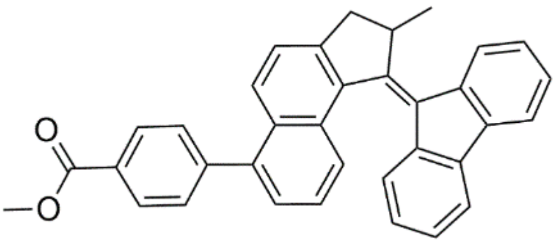

A mixture of **M8** (1.27 g, 3.0 mmol), 4-(methoxycarbonyl)benzeneboronic acid (576 mg, 3.2 mmol), Ag_2O (69 mg, 0.3 mmol), Cs_2CO_3 (5.86 g, 18.0 mmol), $Pd(PPh_3)_4$ (347 mg, 0.3 mmol) was stirred in dry and degassed THF (50 mL) with Ar_2 at 80°C for 24 h. The mixture

was allowed to cool down to room temperature, after which it was diluted with diethyl ether (30 mL) and filtrated. After removal of the solvent, the residue was purified by column chromatography (SiO_2, pentane: CH_2Cl_2 = 5:1) to yield **M1** as yellow solid (789 mg, 55%). ^{1}H NMR (400 MHz, $CDCl_3$): δ = 8.21 (d, J = 8.5 Hz, 2H), 8.06 – 7.96 (m, 1H), 7.92 (d, J = 8.5 Hz, 1H), 7.89 – 7.82 (m, 2H), 7.77 (d, J = 7.5 Hz, 1H), 7.66 (d, J = 8.1 Hz, 2H), 7.53 (d, J = 8.5 Hz, 1H), 7.41 (dd, J = 5.8, 3.0 Hz, 3H), 7.38 – 7.31 (m, 1H), 7.23 (t, J = 7.2 Hz, 1H), 6.86 (t, J = 7.1 Hz, 1H), 6.80 (d, J = 7.8 Hz, 1H), 4.46 – 4.30 (m, 1H), 3.99 (s, 3H), 3.58 (dd, J = 15.1, 5.6 Hz, 1H), 2.76 (d, J = 15.1 Hz, 1H), 1.40 (d, J = 6.7 Hz, 3H); ^{13}C NMR (100 MHz, $CDCl_3$): δ = 167.09, 151.08, 147.45, 145.99, 140.15, 139.85, 139.80, 139.66, 137.10, 136.84, 130.78, 130.47, 130.38, 130.28, 129.62, 129.12, 128.57, 127.71, 127.04, 126.48, 126.03, 125.76, 124.36, 124.18, 119.72, 119.03, 52.24, 45.64, 41.92, 19.29. HRMS (ESI) calcd for $C_{35}H_{26}O_2Na^+$ 501.1829, found 501.1858. Anal Calcd for $C_{35}H_{26}O_2$: C 87.84, H 5.48, O 6.69; found: C 87.73, H 5.71, O 6.56. Chiral resolution of **M1** was achieved by preparative HPLC, using a chiral ChiralCN OD column (Guangzhou Research & Creativity Biotechnology Co., Ltd.) in hexane: EtOH = 3:7, the enantiomeric excess (*e.e.*) = 99.0 %.

3. Kinetic analysis of motors M1-M3 in THF

The thermal relaxation rate (k_Δ) of the first-order thermal motion of the motors **M1-M3** (40 μM in THF) was obtained by the decrease in absorbance at 450 nm at 20 0 °C, 25 °C, 30 °C and 35 °C, fitting the first-order chemical reaction:

$$A(t) = A_\infty - (A_\infty - A_0)e^{-k_\Delta t} \quad (1)$$

where A_0 is the absorbance at the PSS, A_∞ is the absorbance at the initial state and $A(t)$ is the absorbance at time t in the thermal inversion. Calculations of half-life ($t_{1/2}$), Gibbs free energy of activation ($\Delta^‡G°$), enthalpy of activation ($\Delta^‡H°$) and entropy of activation ($\Delta^‡S°$) were performed using the following equations:

$$t_{1/2} = \frac{\ln 2}{k_{\Delta}} \tag{2}$$

$$\ln\frac{k_{\Delta}h}{k_{B}T} = -\frac{\Delta^{\neq}G^{\circ}}{RT} = -\frac{\Delta^{\neq}H^{\circ} - T\Delta^{\neq}S^{\circ}}{RT} \tag{3}$$

where h is Plank's constant, k_B is Bolzmann constant, T is absolute temperature, R is gas constant. The thermal kinetic parameters are listed in Table S1.

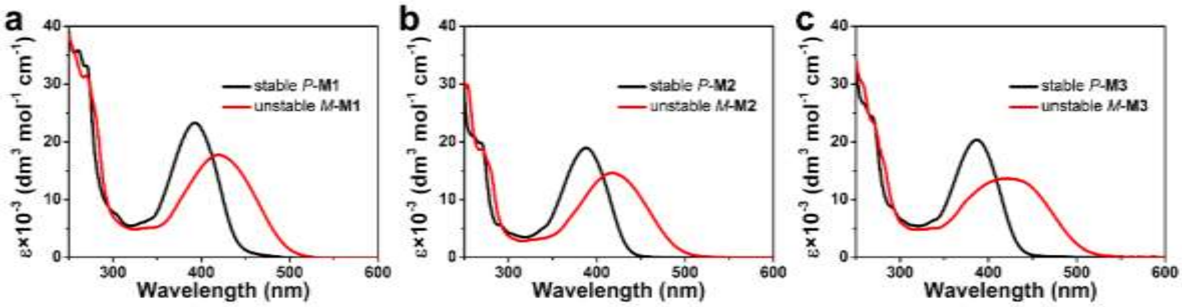

Figure S1. UV-vis absorption spectra of motors in the stable (*P*) form (black) and unstable (*M*) form (red) at room temperature (40 μM in THF): (a) motor **M1**, (b) motor **M2** and (c) motor **M3**.

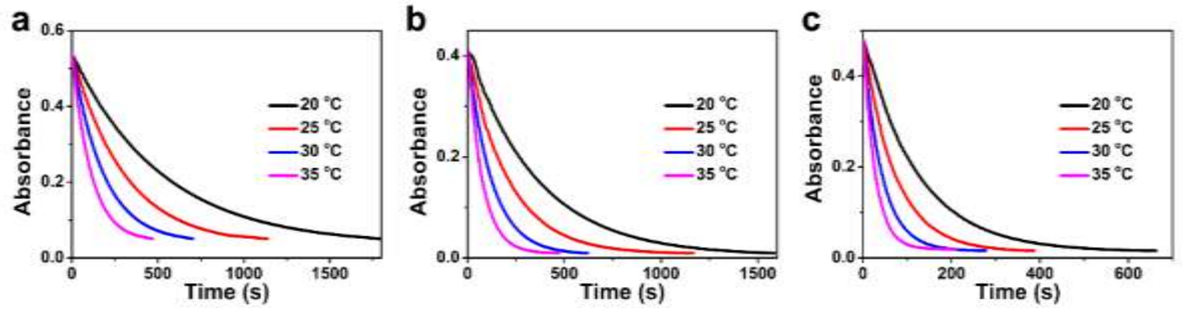

Figure S2. Changes in UV-vis absorption spectra of (a) motor **M1**, (b) motor **M2** and (c) motor **M3** in THF (40 μM) during thermodynamic inversion at 450 nm at different temperature (20 °C, 25 °C, 30 °C, 35 °C).

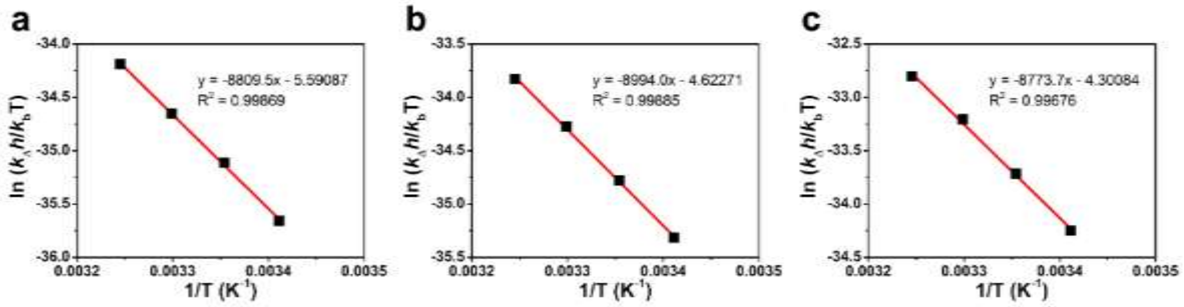

Figure S3. Erying plots for the thermal isomerization from unstable form to stable form in THF: (a) motor **M1**, (b) motor **M2** and (c) motor **M3**.

4. Photo-induced and thermo-induced chiroptical variation of chiral motors M1-M3 in cholesteric liquid crystals (CLCs)

The chiroptical variation of chiral motors M1-M3 was measured by Grandjean-Cano wedge method.[3] The CLC mixture was fabricated by doping 1.0 wt% chiral motors into a commercial nematic liquid-crystal host SLC1717. The resulting mixture was capillary filled into a wedge cell (EHC, KCRK-07, tan θ = 0.0196). The wedge cell with an opening angle θ was made by two parallel-alignment substrates with two spacers in different thickness at each end of the cell (Figure S4). The parallel arrangement of CLC produces the disclination lines between the CLC layers in which the interlayer spacer is integer multiples of $p/2$, where p is the pitch of CLCs as shown in Figure S3. The disclination lines can be observed by a transmission-mode POM. Therefore the pitch can be calculated by the equation $p = 2L \tan \theta$, where L is the distance between the disclination lines.

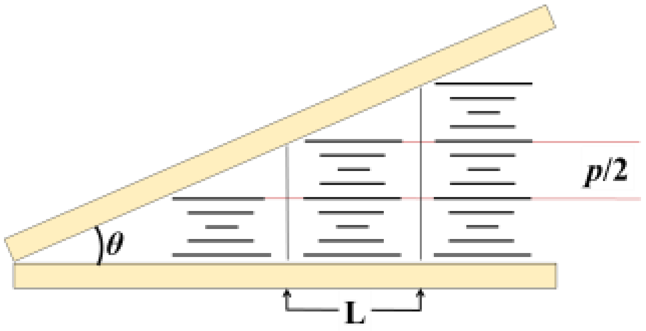

Figure S4. Schematic illustration of a Grandjean-Cano wedge cell for measuring the pitch of CLC.

Upon UV exposure (365 nm, 20.0 mW cm^{-2}) on the CLCs containing **M1** (1.0% wt%) in wedge cells, the distance of the disclination lines rapidly shortened during the photochemical reaction (Figure S5a), whereas the distance of the disclination lines in the CLCs containing **M2** (1.0% wt%) or **M3** (1.0 wt%) expanded beyond the black field range (unwinding CLCs, chiral handedness inversion), and then the disclination lines reappeared with chiral handedness inversion as well as closed up each other until the PSS (Figures S5b and S5c). After terminating UV light, the three motor-doped CLCs inversed to their initial states along

with the thermodynamic conversion. The helical twisting power (HTP, denoted as β) of chiral motors was then determined by the equation $\beta = 1/(pc)$, where the positive and negative sign represent right-handed (RH) and left-handed (LH) helix, respectively, and c is the concentration of the chiral motor.

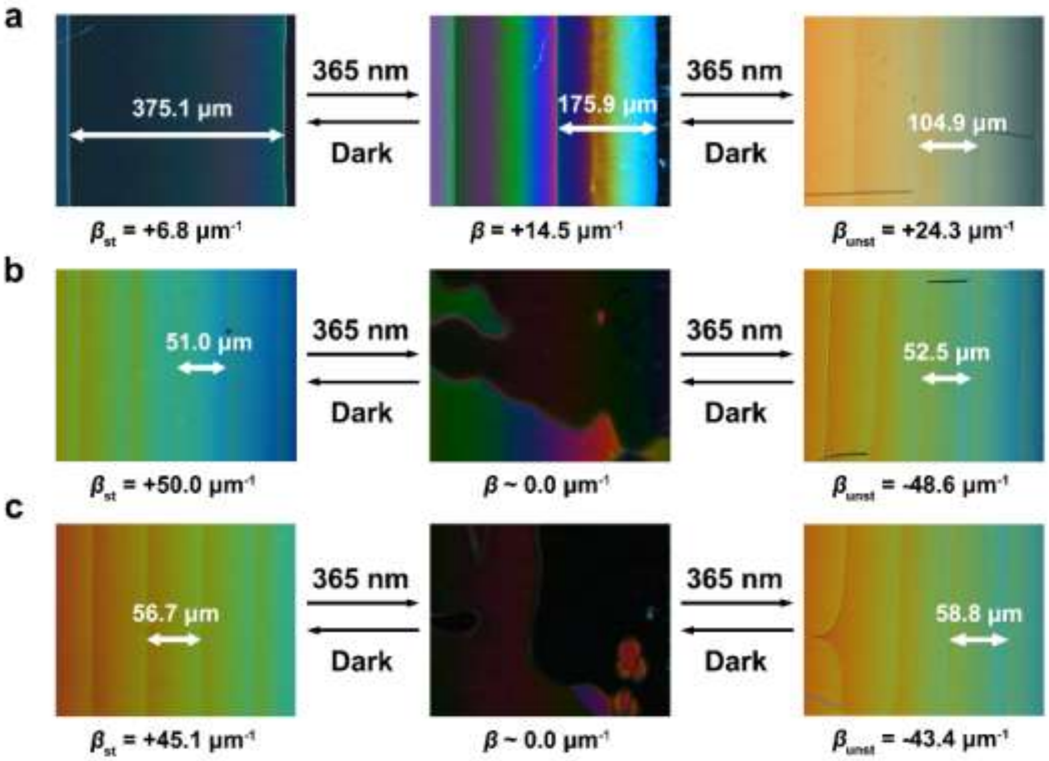

Figure S5. Dynamic chiroptical behavior of CLC mixtures (1.0 wt% chiral motor in SLC1717) in the wedge cell upon UV light exposure (365 nm, 20.0 mW cm^{-2}) and dark relaxation observed by a transmission-mode POM: (a) motor **M1**, (b) motor **M2** and (c) motor **M3**.

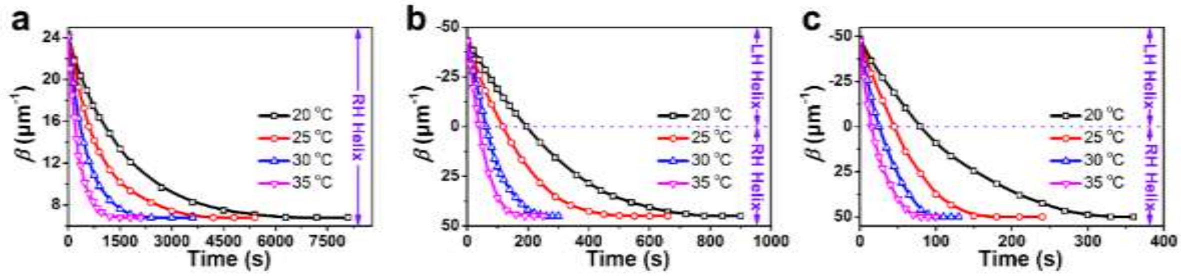

Figure S6. HTP change of (a) motor **M1**, (b) motor **M2** and (c) motor **M3** (1.0 wt% in SLC1717) during thermodynamic inversion at different temperature, respectively.

5. Dynamic analysis of chiral motors M1-M3 in CLCs

The k_Δ of the motors **M1-M3** (1.0 wt% in SLC1717) was obtained by following the molar fraction of *M* form (χ_{unst}) with respect to time (t) 20 0 °C, 25 °C, 30 °C and 35 °C, fitting the first-order chemical reaction:

$$\chi_{unst}(t) = \chi_{unst,PSS}e^{-k_\Delta t} \quad (4)$$

where $\chi_{unst,PSS}$ is the molar fraction of *M* form at the PSS, normalizing $\chi(0) = 1$ and $\chi(\infty) = 0$ at 20 °C. Calculations of $t_{1/2}$, $\Delta^{\ddagger}G°$, $\Delta^{\ddagger}H°$ and $\Delta^{\ddagger}S°$ were also performed using the Erying equations (2) and (3). The thermal kinetic parameters are listed in Table S1.

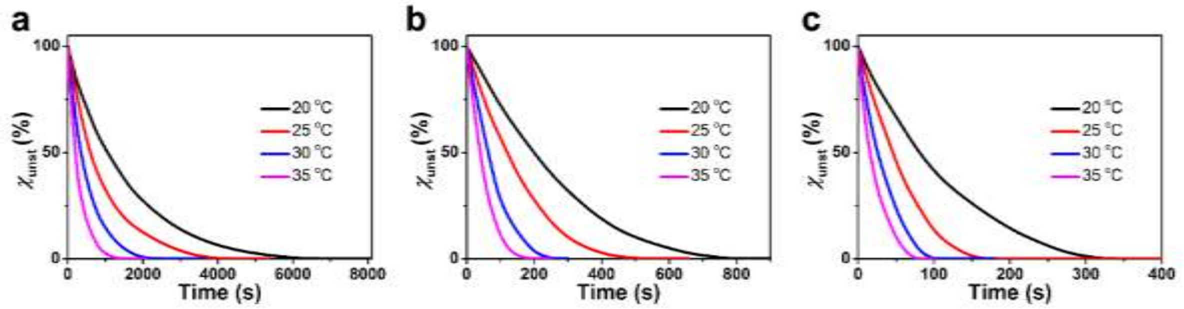

Figure S7. Changes in molar ratio of χ_{unst} of (1) motor **M1**, (2) motor **M2** and (3) motor **M3** (1.0 wt% in SLC1717) during thermal relaxation at different temperature (20 °C, 25 °C, 30 °C, 35 °C).

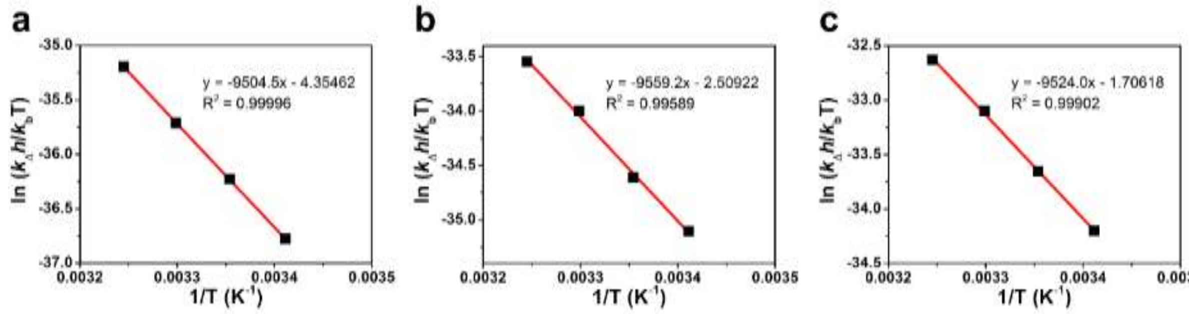

Figure S8. Erying plots for the thermal isomerization from unstable form to stable form in CLC: (a) motor **M1**, (b) motor **M2** and (c) motor **M3**.

Table S1. Thermal Kinetic Parameters of Motors M1, M2 and M3 in Different Media at 20 °C

Media	Motor	k_Δ (s^{-1})	$t_{1/2}$ (s)	$\Delta^{\ddagger}G°$ (kJ mol^{-1})	$\Delta^{\ddagger}H°$ (kJ mol^{-1})	$\Delta^{\ddagger}S°$ (J mol^{-1} K^{-1})
THF	**M1**	2.0×10^{-3}	347	85.3	71.7	-46.5
	**M2**	2.8×10^{-3}	248	84.5	71.7	-43.4
	**M3**	8.2×10^{-3}	85	81.8	71.4	-35.8

	**M1**	6.5×10^{-4}	1064	87.9	77.3	-36.2
LC	**M2**	3.5×10^{-3}	198	83.9	77.8	-20.9
	**M3**	8.6×10^{-3}	81	81.6	77.5	-14.2

6. Fabrication and characterizations of motor-doped CLCs and polymer-stabilized CLCs (PSCLCs)

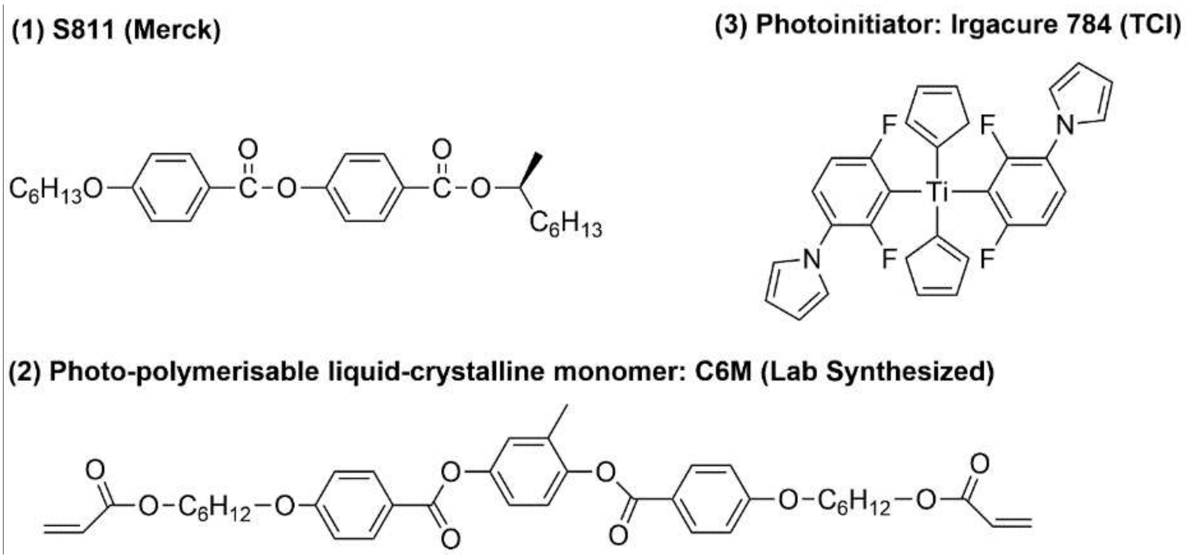

Figure S9. Chemical structures of components utilized in the present study: (1) chiral dopant S811, (2) photo-polymerisable liquid-crystalline monomer C6M and (3) photoinitiator Irgacure 784.

Table S2. Composition of the photoresponsive motor-doped CLCs and PSCLCs

Mixture	Motor	Composition of the CLC (wt%)				
		Motor	S811	SLC1717	C6M	Irgacure 784
**M1**-doped CLC	**M1**	8.5	0.8	90.7	/	/
**M2**-doped CLC	**M2**	1.5	12.5	86.0	/	/
**M3**-doped CLC	**M3**	1.7	12.5	85.8	/	/
**M1**-doped PSCLC	**M1**	8.5	0.8	69.7	20.0	1.0
**M2**-doped PSCLC	**M2**	1.5	12.5	65.0	20.0	1.0
**M3**-doped PSCLC	**M3**	1.7	12.5	64.8	20.0	1.0

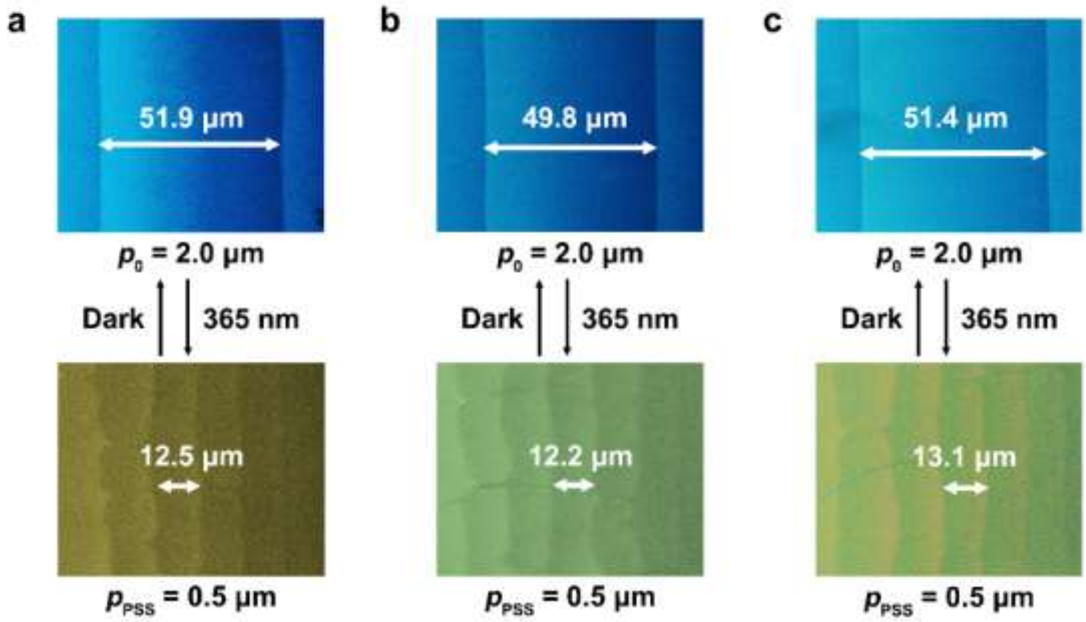

Figure S10. POM images and helical pitch variation of motor-doped CLCs in the wedge cell upon UV light exposure (365 nm, 20.0 mW cm^{-2}) and dark relaxation: (a) **M1**-doped CLC, (b) **M2**-doped CLC and (c) **M3**-doped CLC.

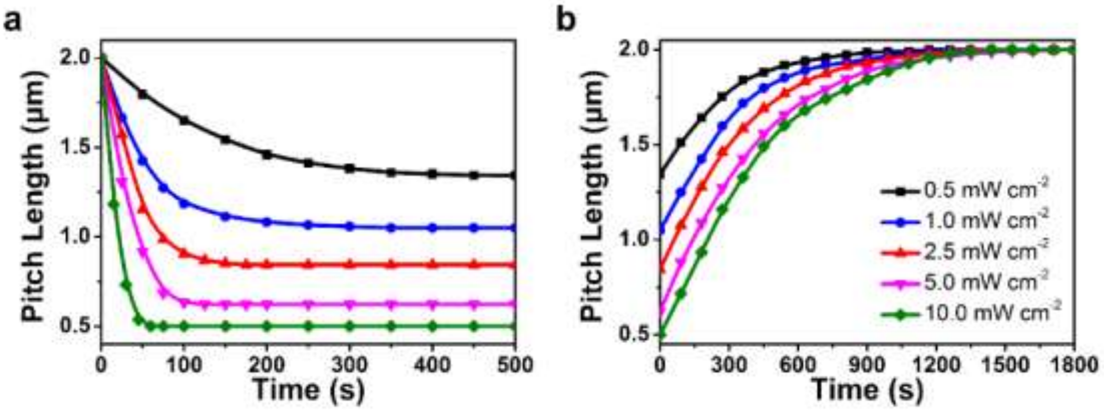

Figure S11. Dynamic variation of helical pitch of **M1**-doped CLC in the wedge cell under (a) under different UV-exposure intensities (0.5, 1.0, 2.5, 5.0 and 10.0 mW cm^{-2}) and (b) dark relaxation.

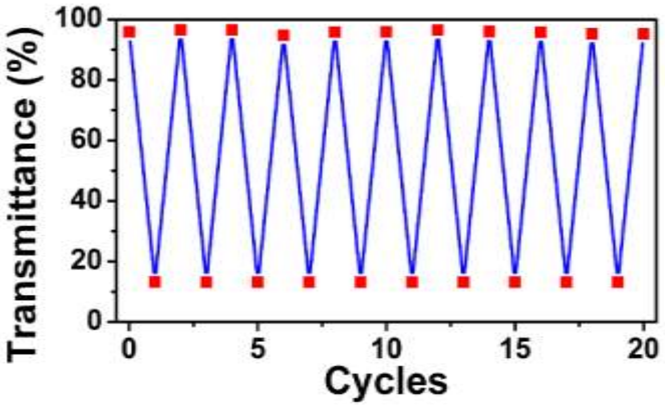

Figure S12. Dynamic cycles of transmittance of **M1**-doped CLC at 633 nm with UV light for 120 s and then dark relaxation for 1800 s repeatedly.

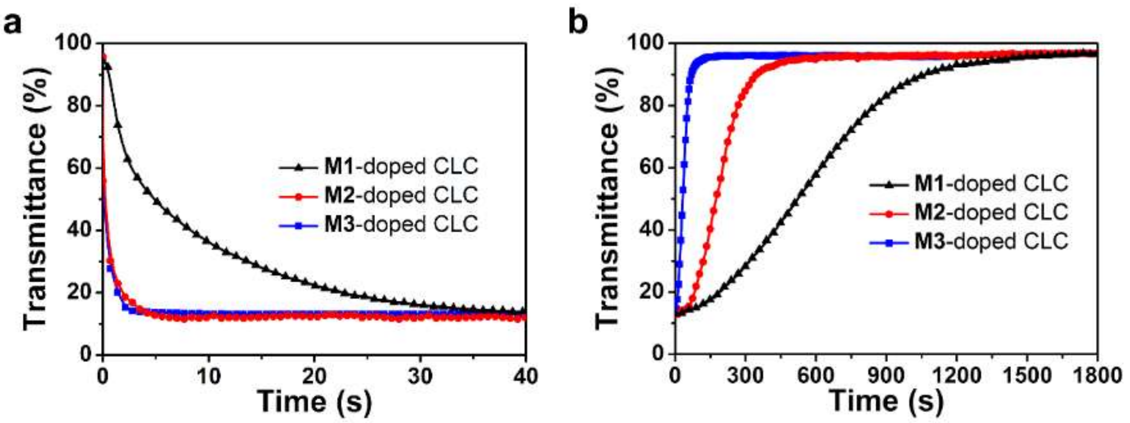

Figure S13. (a) Photodynamic and (b) thermodynamic transmittance measurement (633 nm) at room temperature: **M1**-doped CLC, **M2**-doped CLC and **M3**-doped CLC.

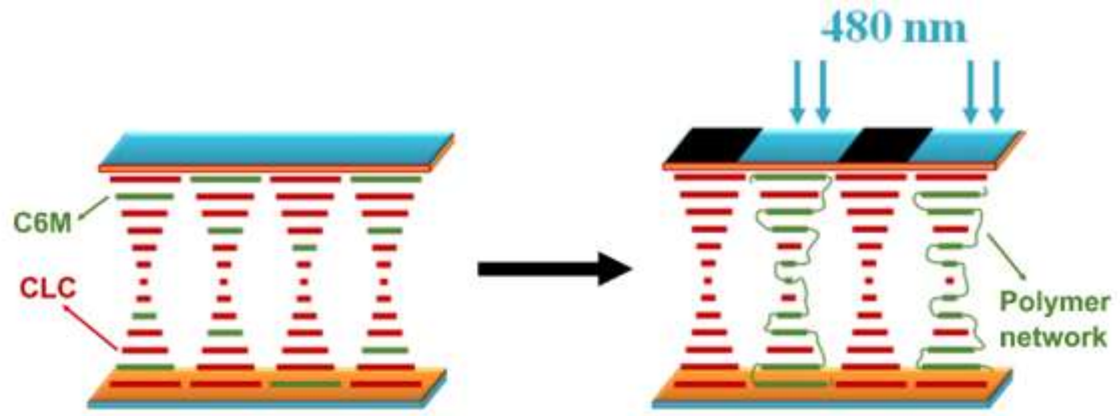

Figure S14. Schematic representation for fabrication of the diffraction grating CLCs.

To fabricate the diffraction grating CLCs, the motor-doped CLCs containing C6M (20.0 wt%) and Irgacure 784 (1.0 wt%), as listed in Table S2, were homogeneously mixed at 80 ^{o}C, and then capillary-filled into the 15 μm-thick cells treated with anti-parallel alignment at room temperature. Upon exposure to a 480 nm laser (5.0 mW cm^{-2}) though the photomask for 30 min at 40 ^{o}C, the C6M monomers were trigger by free radical polymerization to form the liquid-crystalline polymer networks with the arrangement of CLCs. After photo-polymerization, the exposed and unexposed areas exhibit unapparent difference in the cells.

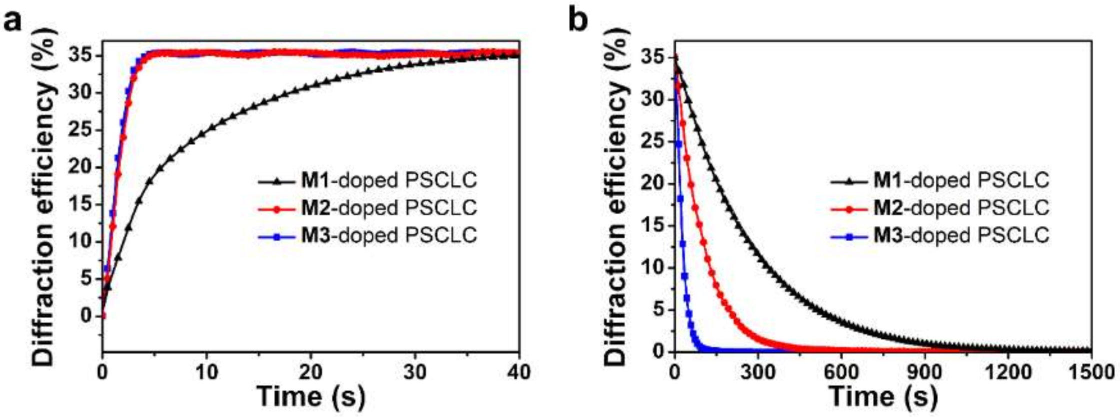

Figure S15. (a) The rise and (b) decay response time of first-order diffraction efficiency for **M1**-doped PSCLC, **M2**-doped PSCLC and **M3**-doped PSCLC gratings.

7. ^{1}H NMR and ^{13}C NMR spectra

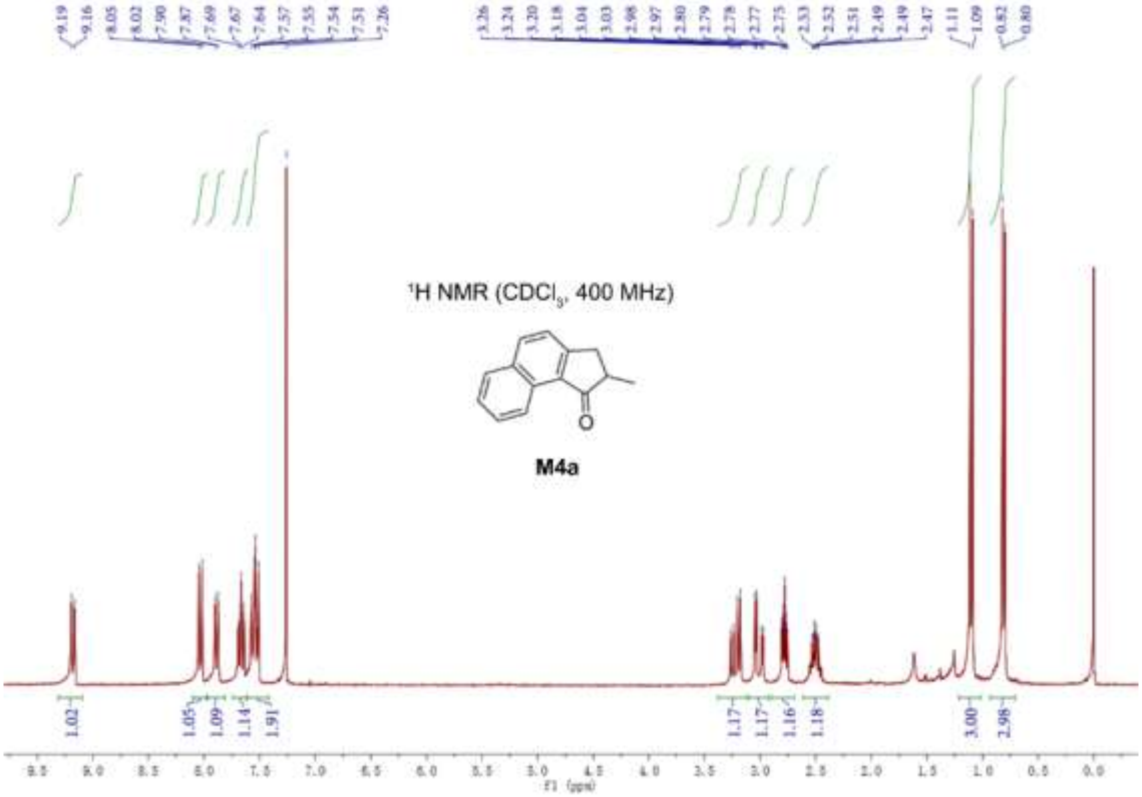

Figure S16. ^{1}H NMR spectrum of M4a.

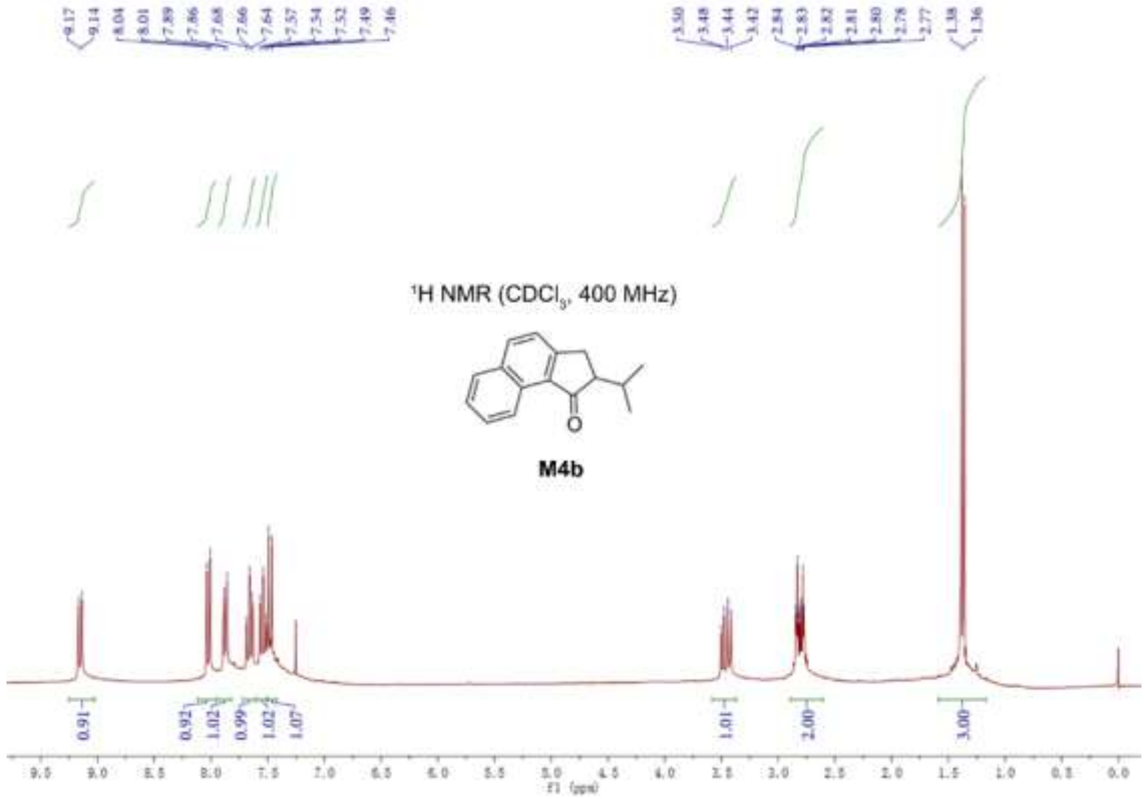

Figure S17. ^{1}H NMR spectrum of M4b.

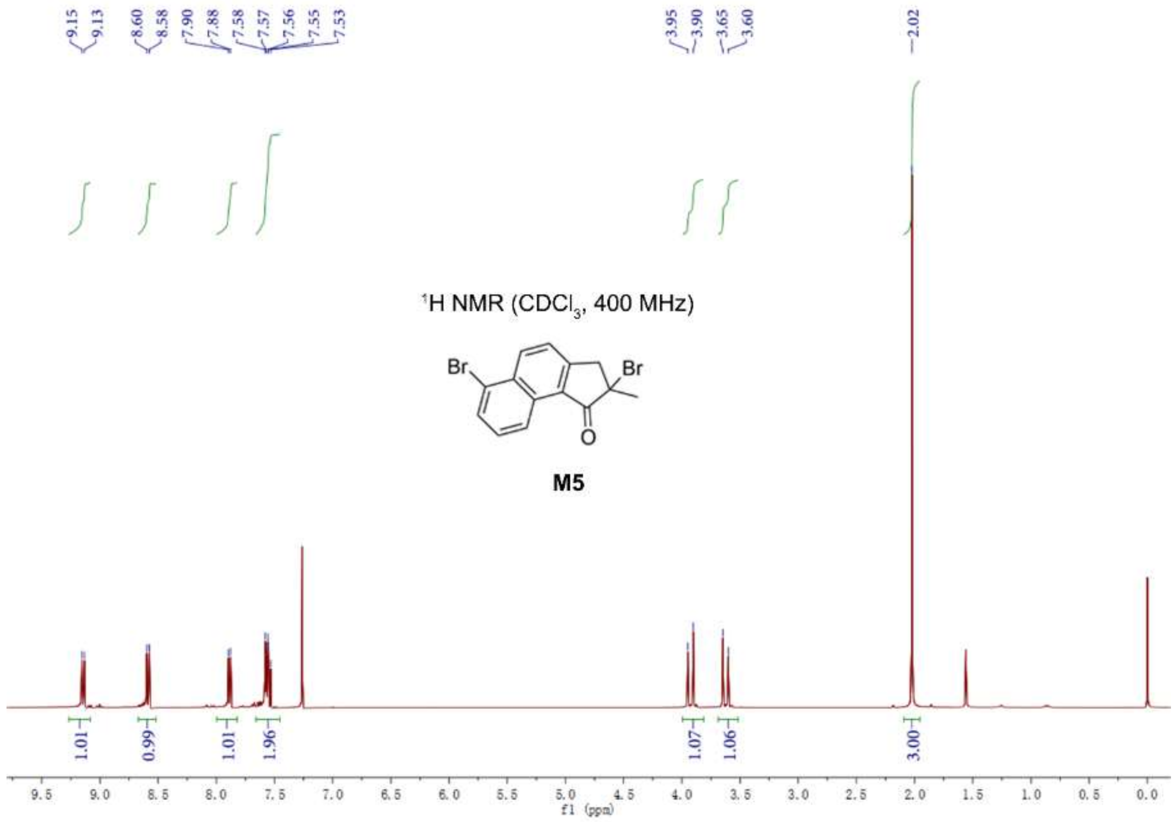

Figure S18. ^{1}H NMR spectrum of M5.

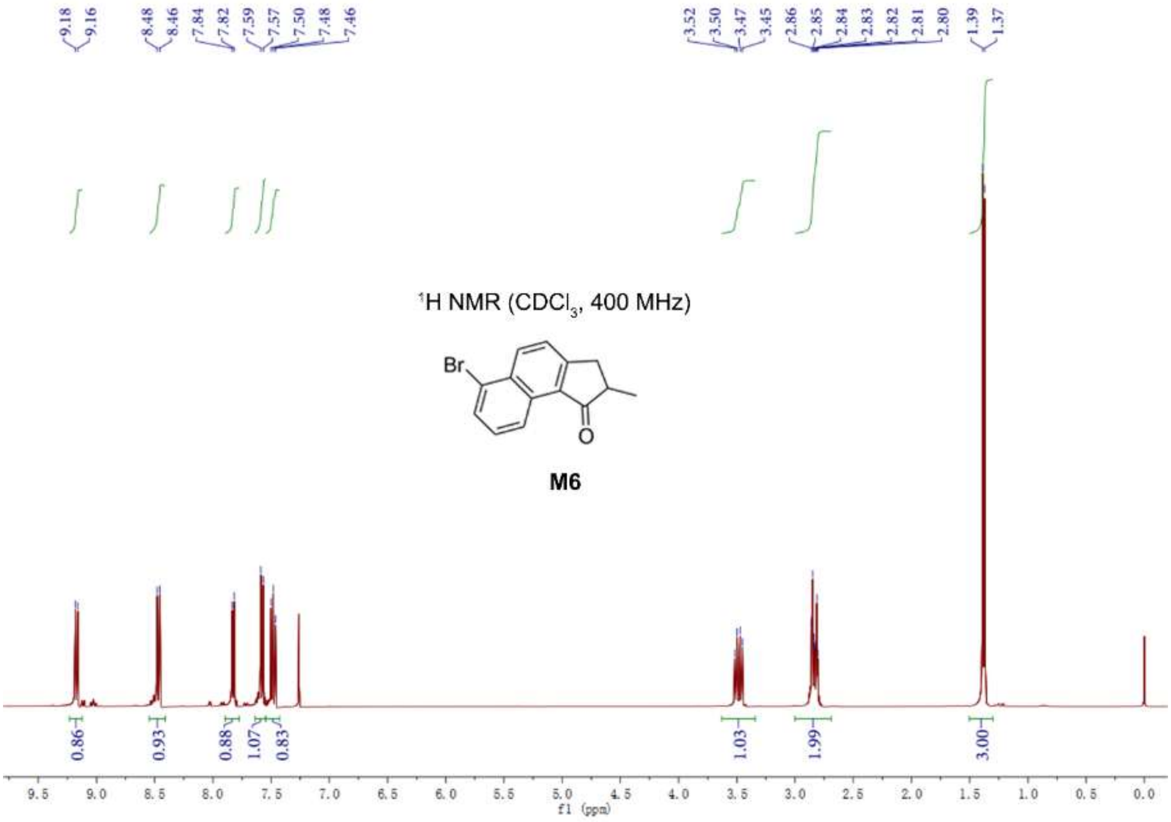

Figure S19. ^{1}H NMR spectrum of M6.

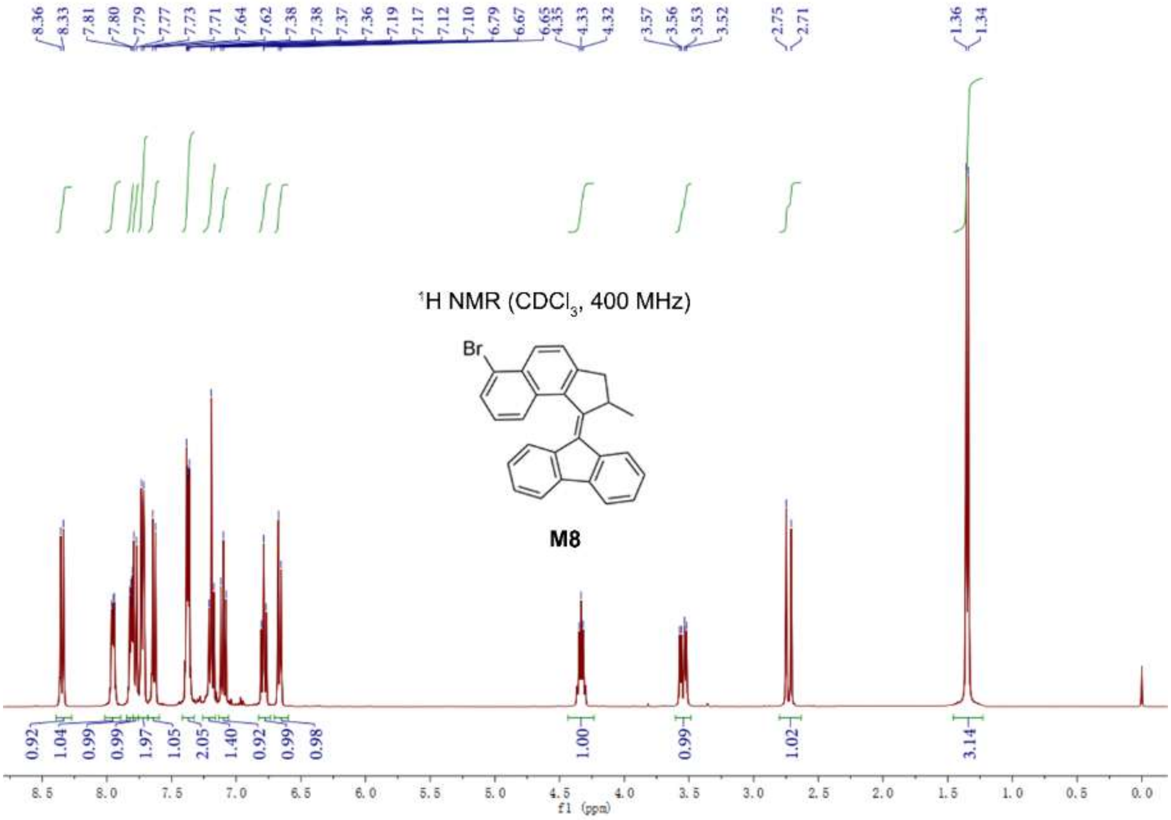

Figure S20. ^{1}H NMR spectrum of M8.

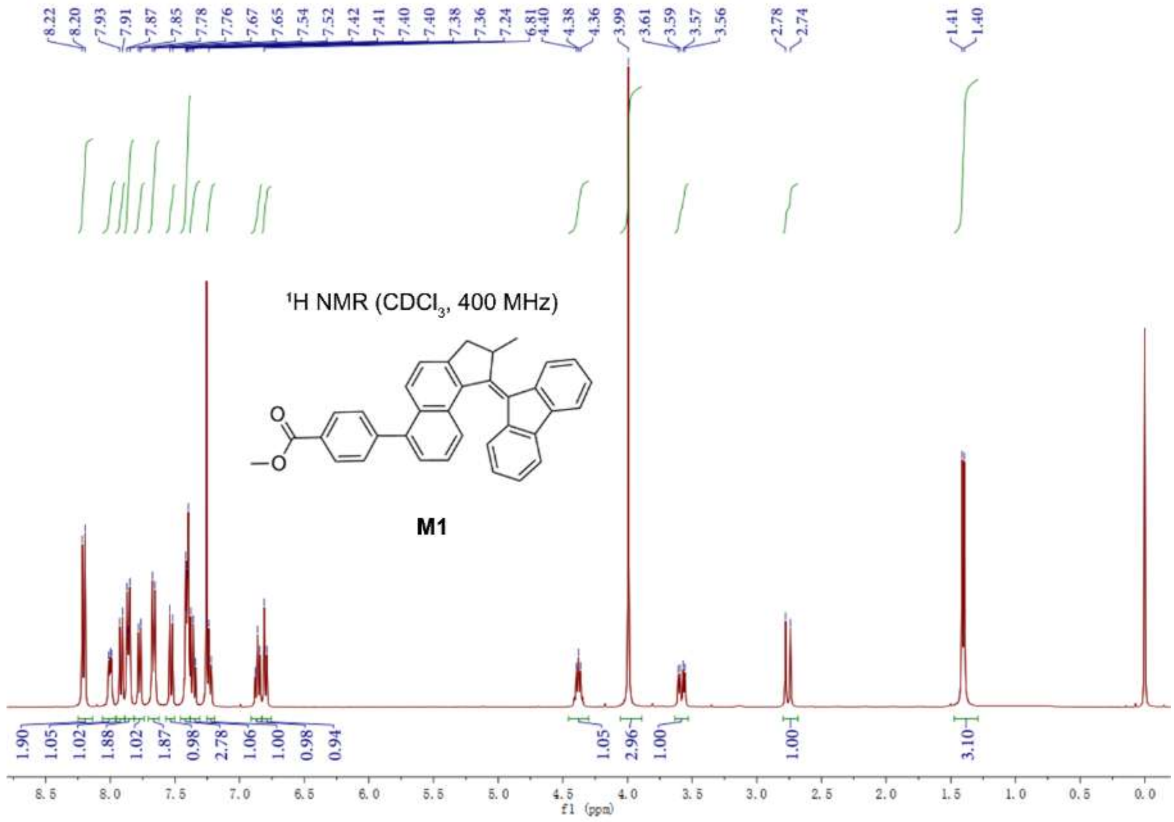

Figure S21. ^{1}H NMR spectrum of M1.

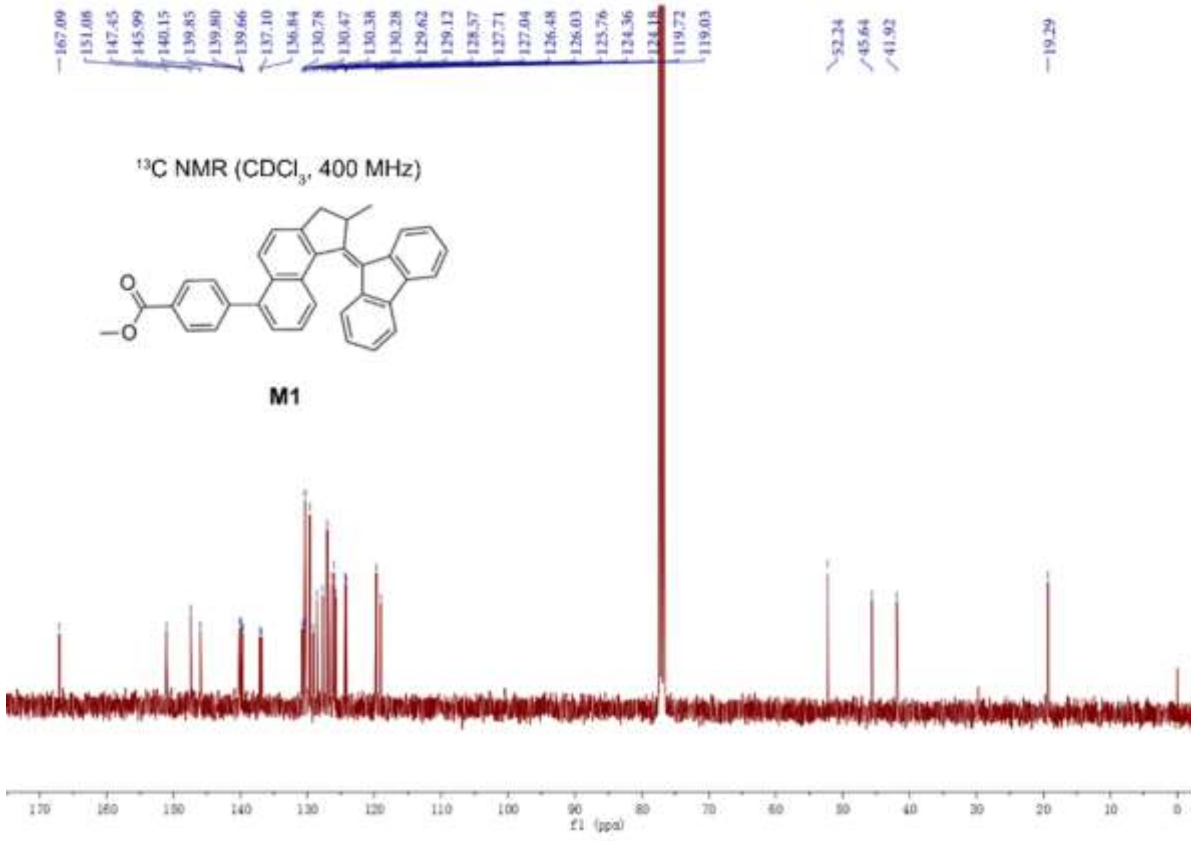

Figure S22. ^{13}C NMR spectrum of M1.

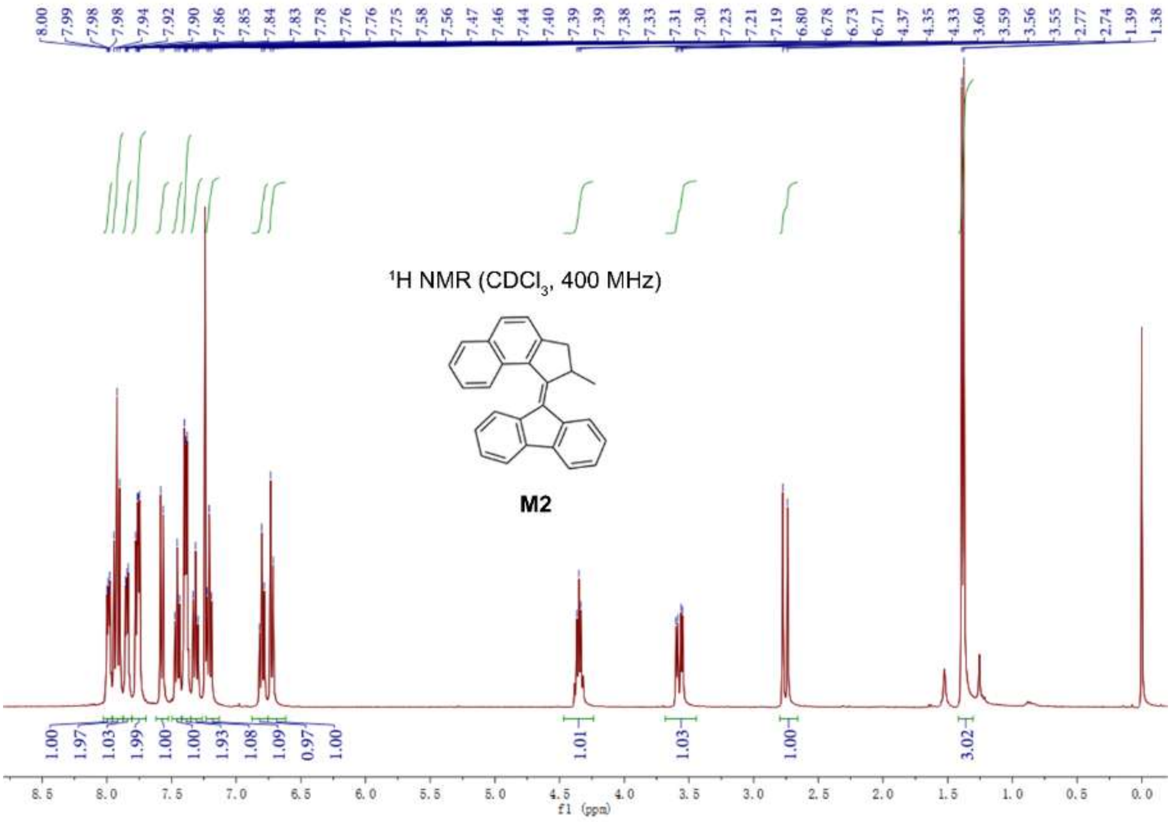

Figure S23. ^{1}H NMR spectrum of M2.

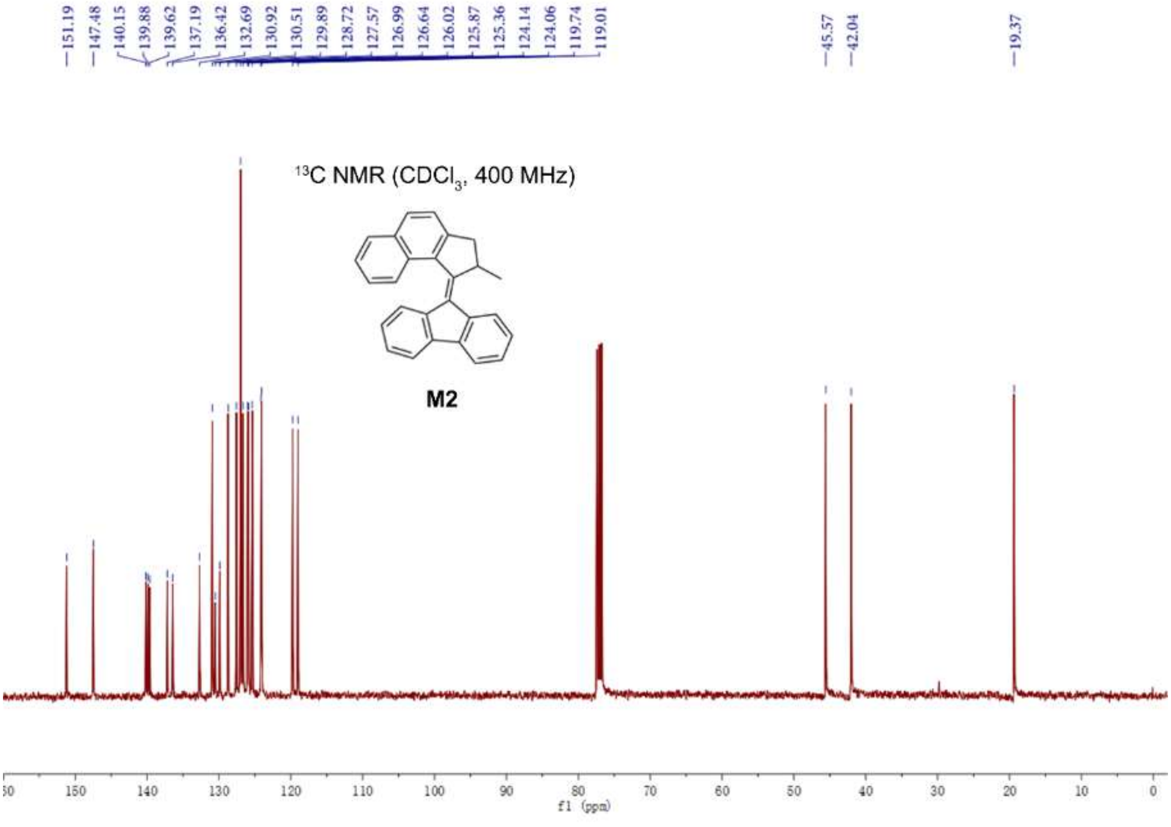

Figure S24. ^{13}C NMR spectrum of M2.

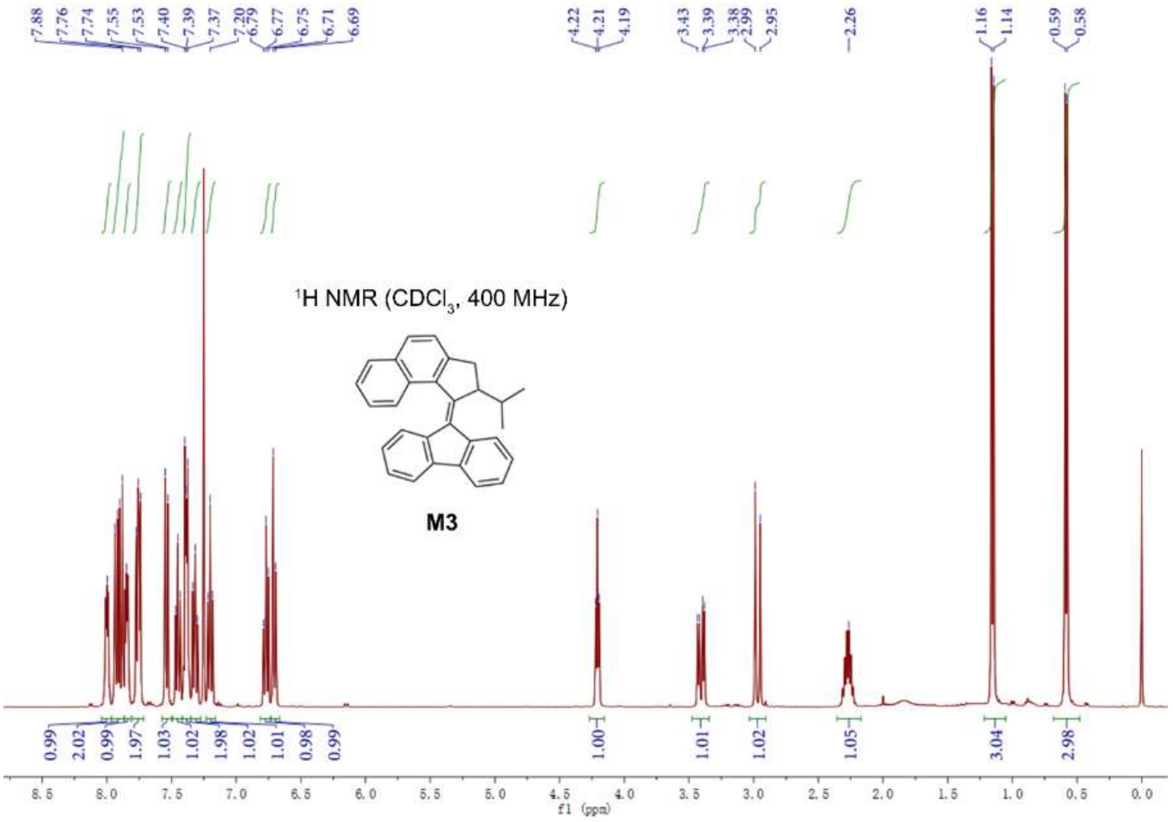

Figure S25. ^{1}H NMR spectrum of M3.

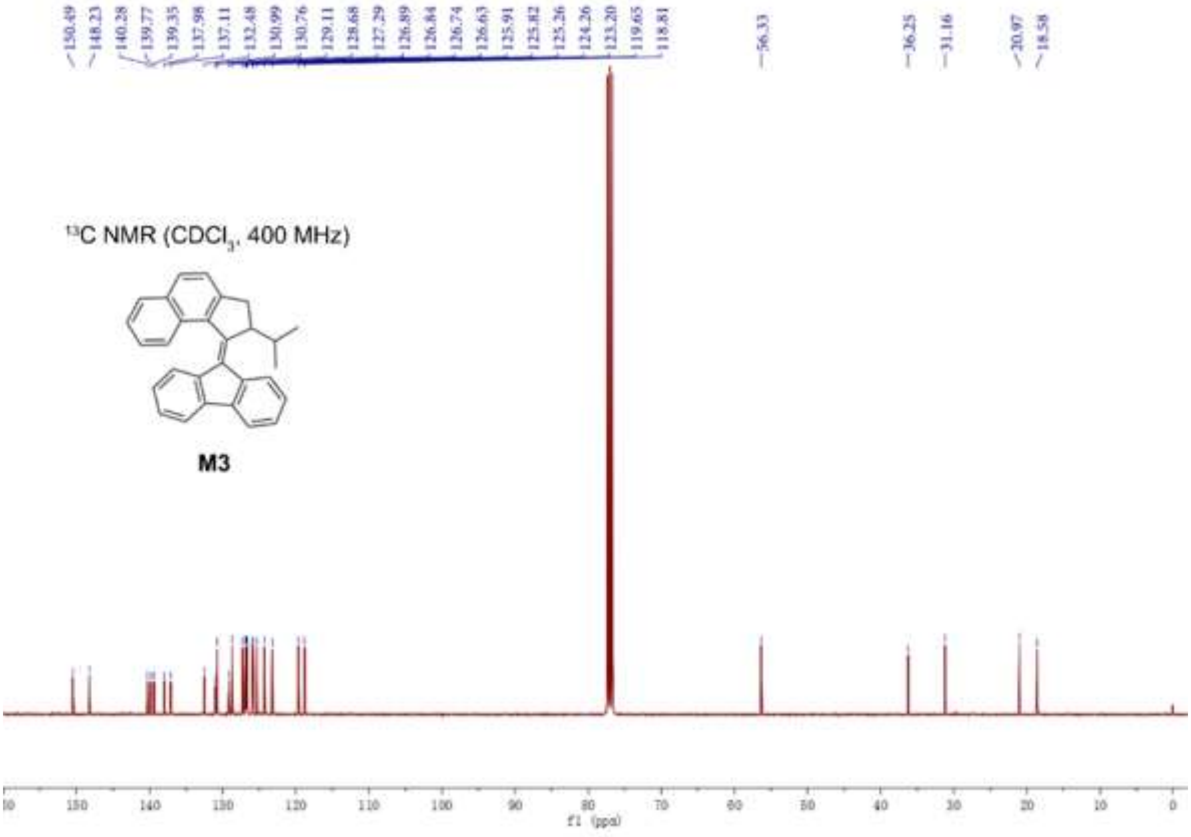

Figure S26. ^{13}C NMR spectrum of M3.

8. References

(1) Hu, W.; Zhao, H.; Song, L.; Yang, Z.; Cao, H.; Cheng, Z.; Liu, Q.; Yang, H. *Adv. Mater.* **2010**, *22*, 468.

(2) Chen, J.; Kistemaker, J. C. M.; Robertus, J.; Feringa, B. L. *J. Am. Chem. Soc.* **2014**, *136*, 14924.

(3) Dierking, I. *Textures of Liquid Crystals*; Wiley: Weinheim, 2003.
